# Comparative Anatomical and Transcriptomics Reveal the Larger Cell Size as a Major Contributor to Larger Fruit Size in Apricot

**DOI:** 10.3390/ijms24108748

**Published:** 2023-05-14

**Authors:** Mengzhen Huang, Xuchun Zhu, Haikun Bai, Chu Wang, Ningning Gou, Yujing Zhang, Chen Chen, Mingyu Yin, Lin Wang, Tana Wuyun

**Affiliations:** 1 State Key Laboratory of Tree Genetics and Breeding, Research Institute of Non-Timber Forestry, Chinese Academy of Forestry, Zhengzhou 450003, China; mengzhen4524@163.com (M.H.); xxcy08@163.com (X.Z.); bhk1994@163.com (H.B.); wangchu1226@163.com (C.W.); lemonn@caf.ac.cn (N.G.); zhangyujing@caf.ac.cn (Y.Z.); chenchenbo@caf.ac.cn (C.C.); ymy920916@163.com (M.Y.); wanglin1815@163.com (L.W.); 2 College of Forestry, Nanjing Forestry University, Nanjing 210037, China; 3 Kernel-Apricot Engineering and Technology Research Center of State Forestry and Grassland Administration, Zhengzhou 450003, China; 4 Key Laboratory of Non-Timber Forest Germplasm Enhancement and Utilization of National Forestry and Grassland Administration, Zhengzhou 450003, China

**Keywords:** apricot, fruit size, cell size, RNA-seq, auxin signal transduction, expansin, PRE6

## Abstract

Fruit size is one of the essential quality traits and influences the economic value of apricots. To explore the underlying mechanisms of the formation of differences in fruit size in apricots, we performed a comparative analysis of anatomical and transcriptomics dynamics during fruit growth and development in two apricot cultivars with contrasting fruit sizes (large-fruit *Prunus armeniaca* ‘Sungold’ and small-fruit *P. sibirica* ‘F43’). Our analysis identified that the difference in fruit size was mainly caused by the difference in cell size between the two apricot cultivars. Compared with ‘F43’, the transcriptional programs exhibited significant differences in ‘Sungold’, mainly in the cell expansion period. After analysis, key differentially expressed genes (DEGs) most likely to influence cell size were screened out, including genes involved in auxin signal transduction and cell wall loosening mechanisms. Furthermore, weighted gene co-expression network analysis (WGCNA) revealed that *PRE6/bHLH* was identified as a hub gene, which interacted with 1 *TIR1*, 3 *AUX/IAAs*, 4 *SAURs*, 3 *EXPs,* and 1 *CEL*. Hence, a total of 13 key candidate genes were identified as positive regulators of fruit size in apricots. The results provide new insights into the molecular basis of fruit size control and lay a foundation for future breeding and cultivation of larger fruits in apricot.

## 1. Introduction

Fruits, a unique reproductive organ of angiosperms, are responsible for providing the necessary nutrients for human beings and livestock, including vitamins, carbohydrates, fibers, and trace elements, in addition to being able to disperse seeds [1,2]. Larger-size or higher-weight fruits have long been an important and predominantly selection target for humans during plant domestication and improvement [3,4,5] because it usually brings higher quality, yield, and better consumer acceptance to obtain greater economic benefits. Although it is well known that one or more factors interact to influence fruit size, such as genetics, environments, and cultivation practices [6,7,8], revealing the genetic basis and molecular mechanisms that control fruit size remain crucial and elusive because of the complex process of fruit growth and development [2,9]. Many studies have identified that the three major developmental processes determine fruit growth, development, and final fruit size, consisting of cell division, endoreduplication, and cell expansion [10,11,12]. 

Research on fruit size or weight has made important progress in several model species for a long time, such as *Arabidopsis thaliana* [13], *Oryza sativa* [14], *Solanum lycopersicum* [2], and *Cucumis sativus* [15,16]. In addition, several molecular mechanisms have been recognized as major factors to coordinately regulate fruit development and fruit size [17,18], including phytohormones [9,19], transcription factors [20], quantitative trait loci (QTLs) [7,15], the CLV-WUS signaling pathway [21], the ubiquitin–proteasome pathway [22], microRNA pathways [23,24], etc. Moreover, multiple loci and genes identified for the control of fruit size have relatively conserved functions or are homologous to each other among species [15,25,26], although they may explain phenotypic variation through different molecular mechanisms. For example, *FW2.2* [27] and its homologs *CNR/FWL* have been reported to influence various organ sizes in multiple plant species by regulating cell numbers, such as maize [28], rice [29], soybean [30], and *Physalis floridana* [31], even though their precise function and true mechanisms in controlling cell division remain to be deciphered [32]. Concretely, a previous study indicates that *PfCNR1* can interact with *PfAG2* to affect the expression of the *PfCYCD2;1* under certain conditions [31], whereas a novel insight has been proposed that the *MdSIZ1* (the SUMO E3 ligase) sumoylates *MdCNR8* to control apple organ size in a recent report [33]. Up to now, with the improvement of sequencing technology and the comprehensive development of multi-omics, key genes controlling fruit size have been constantly excavated and verified, but they are mainly derived from annual crops. However, in perennial fruit tree crops, the identification of key genes controlling fruit size is still a great challenge due to their relatively long juvenile period and the polygenic characteristic of the fruit size trait [34].

Apricot is usually recognized as one of the typical drupe members of the Rosaceae family [35]. Apricot fruits are rich in total soluble solids and vitamins [36,37], and their kernels are full of protein and carbohydrates [38], which makes apricots extremely valuable both economically and nutritionally. In general, the apricot widely cultivated all around the world refers to the species *Prunus armeniaca* L. (also called the ‘common apricot’) [39], although five other distinct apricot species including *P. sibirica* L., *P. mume* (Sieb.) Sieb. and Succ., *P. mandshurica* (Maxim), *P. brigantina* Vill., and *P. holosericeae* Batal are also recognized [39]. Multiple genome sequences [40,41,42] have been reported since the first publication of the *P. armeniaca* (‘Chuanzhihong’) genome [43], which provide an opportunity to elucidate the connection between the genetic diversity and phenotypic variation for important agronomic traits in apricots. Further, the accessibility to genomes offers a more effective reference to explore transcriptional regulatory mechanisms of fruit color [43,44,45] and taste [45] in apricots. Fruit size is one of the important quality traits in apricots [39], and multiple QTLs related to it have been obtained in recent reports [46,47,48]. In contrast, almost no studies have been performed to mine out the transcriptome dynamics underlying fruit size traits in apricots. 

Here, we performed a temporal and spatial analysis of anatomical and transcriptomics in two apricot cultivars with contrasting fruit sizes (large-fruit *P. armeniaca* ‘Sungold’ and small-fruit *P. sibirica* ‘F43’) during the entire fruit growth and development. We dissected these results to reveal the major factor that determines the larger fruit and to identify key candidate genes that regulate fruit size. These results offer a new clue and detailed evidence to understand the molecular basis underlying fruit size in apricots.

## 2. Results

### 2.1. Comprehensive Analysis of the Contribution of Cell Size to Apricot Fruit Size

**Figure 1:**
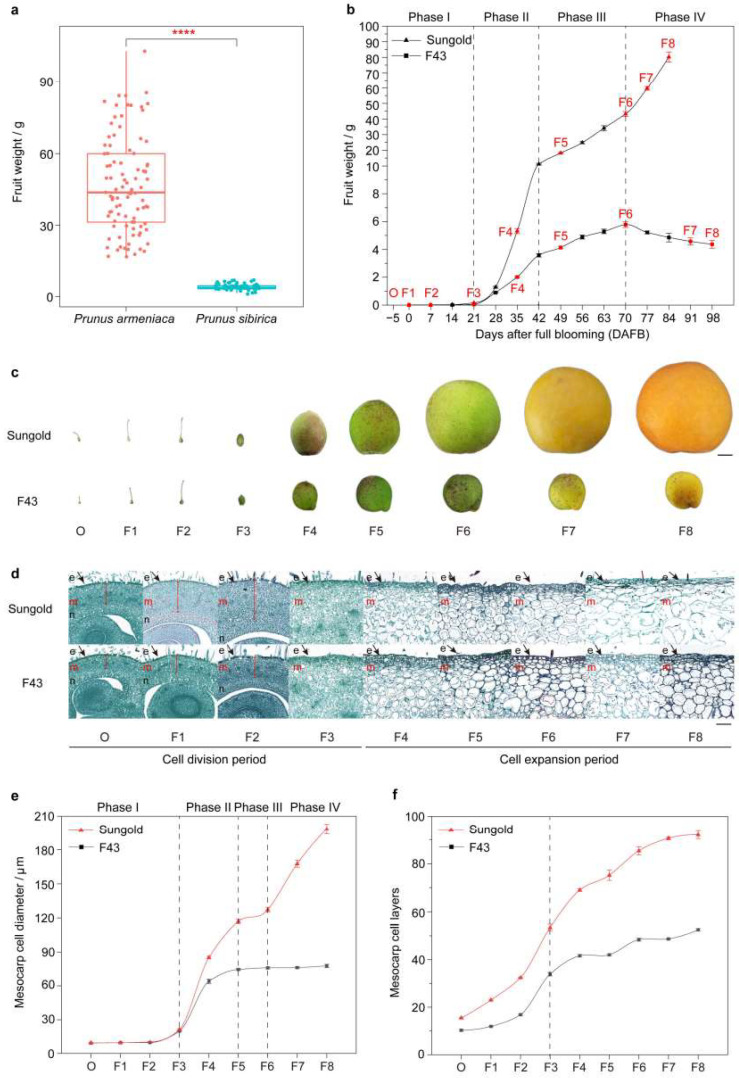
Fruit development and cell growth dynamics of large ‘Sungold’ fruit and small ‘F43’ fruit. (**a**) Fruit weight in *P. armeniaca* and *P. sibirica*. Significance testing was conducted using *T*-test (**** *p* < 2 × 10^−16^). (**b**) The comparison of fruit weight between ‘Sungold’ and ‘F43’ at 13 or 15 time points during development. Values are means ± standard error (*n* = 15). The dashed lines indicate the breakdown of the phases. (**c**) Observations of fruit development of ‘Sungold’ and ‘F43’ at nine representative stages, including ovary 5 days before full blooming (−5, O) and fruit at 0 (F1), 7 (F2), 21 (F3), 35 (F4), 49 (F5), 70 (F6), 77/91 (F7), and 84/98(F8) days after full blooming, bar = 1.0 cm. (**d**) Paraffin sections of mesocarp (cross-sectional of the equatorial position) of ‘Sungold’ and ‘F43’ at nine representative stages, bar = 100 μm. The black arrows indicate the epicarp. The red line segments indicate the mesocarp. e: epicarp; m: mesocarp; n: endocarp. (**e**,**f**) Mesocarp cell diameter and cell layers dynamics of ‘Sungold’ and ‘F43’ at nine representative stages. Values are means ± standard error (*n* = 3). The dashed lines indicate the breakdown of the phases.

In apricots, there is a significant difference in fruit size between *P. armeniaca* and *P. sibirica* (Figure 1a). To understand the reason for the difference in fruit size between larger *P. armeniaca* and smaller *P. sibirica*, ‘Sungold’ and ‘F43’ cultivars were selected to investigate the fruit and cell growth pattern throughout the development process, respectively. Comparisons of fruit weight between ‘Sungold’ and ‘F43’ during the whole development process from the full blooming to the fruit ripening showed that ‘Sungold’ and ‘F43’ exhibited two different growth curves (Figure 1b). According to the differences and similarities between the two growth curves, their development processes were subdivided into four phases: phase Ⅰ (before 21 days after full blooming (DAFB)), phase Ⅱ (between 21 and 42 DAFB), phase III (between 42 and 70 DAFB), and phase IV (after 70 DAFB), respectively. Among them, phases I and III were very similar in the two cultivars and were both relatively flat. However, phase II showed different growth rates, and phase IV exhibited the opposite trend. Particularly, the fruit weight of ‘F43’ reached the peak value at 70 DAFB and subsequently decreased slightly due to the fruit cracking and dehydration during ripening, while the fruit of ‘Sungold’ enlarged a second time at 70 DAFB until fruit maturity. The average fruit weight of ‘F43’ at maturity was only ~4.4 g, while the average fruit weight of ‘Sungold’ was more than 80.0 g. The fruit weight of ‘Sungold’ was ~18.35 times that of ‘F43’ at fruit maturity, and its increased amount in phase IV accounted for ~46.17% of the final fruit weight. The above results indicated that phase IV was a crucial phase affecting the final fruit size in *P. armeniaca*.

Furthermore, a total of nine representative stages from each of ‘Sungold’ and ‘F43’ were selected for paraffin sections, including the ovary 5 days before full blooming (−5, O) and fruit at 0 (F1), 7 (F2), 21 (F3), 35 (F4), 49 (F5), 70 (F6), 77/91(F7), and 84/98 (F8) DAFB (Figure 1b–d). Interestingly, the dynamic curves of fruit mesocarp cell diameter of ‘Sungold’ and ‘F43’ were almost consistent with their respective fruit growth curves, which can be also subdivided into four phases based on the similarities and differences between the two apricots (Figure 1b,e). In phases I and III, the cell diameter did not change much in both kinds of apricots, which were even very close in phase I (Figure 1e). The cell diameter in the two apricots both increased at different rates during phase II, markedly enlarged again in phase IV of ‘Sungold’ and not in ‘F43’ (Figure 1e). The mesocarp cell layers showed a similar changing tendency in the two apricots; all of them reached more than half of their respective final cell layers at stage F3, and then they all slowed their growth (Figure 1f). Thus, according to the comprehensive analysis of the dynamic changes in cell diameter and layers in the two apricots, we divided the cell growth process during fruit development into two parts using ‘Sungold’ as the standard: the cell division period (CDP, stages O, F1-F3) and cell expansion period (CEP, stages F4-F8) (Figure 1d). When the fruits of these two apricot cultivars were ripe, the mesocarp cell layers of ‘Sungold’ was higher by ~0.75-fold than ‘F43’, whereas the mesocarp cell diameter was higher by ~1.55-fold than ‘F43’. Throughout the development process, the cell layers of the two cultivars increased 4.10~4.94-fold from 5 days before full blooming to fruit ripening, but the cell diameter increased ~20.26-fold in ‘Sungold’ and ~7.48-fold in ‘F43’. In addition, cell diameter seemed to be more positively related to fruit weight than cell layers in both apricots, and this relationship was stronger in ‘Sungold’ (Figure S1). As aforementioned, we suggested that the major contribution to the larger fruit size came from the larger cell size in *P. armeniaca*, especially its additional second cell expansion process occurring in phase IV.

### 2.2. Transcriptional Differences between the Two Apricots Concentrated in the Cell Expansion Period during Fruit Development

**Figure 2:**
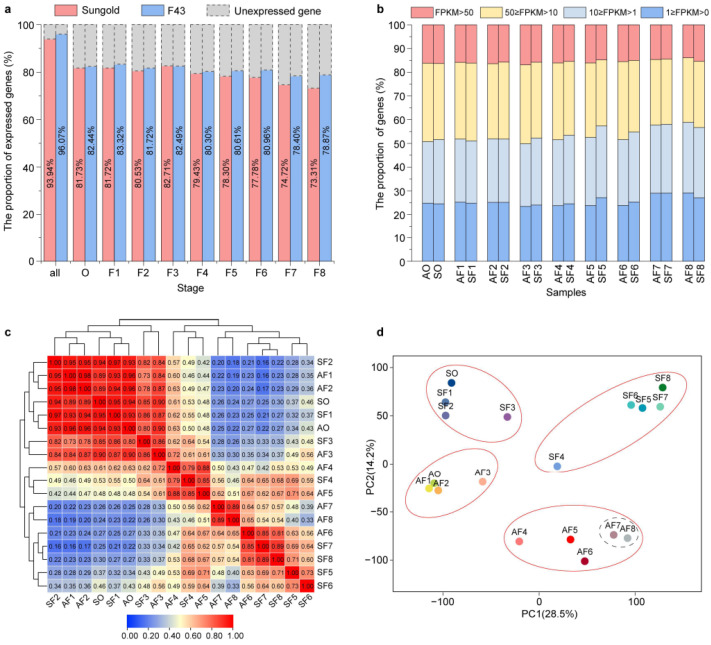
Gene expression and correlation between the transcriptomes of nine representative stages each of two apricot cultivars. (**a**) The proportions of expressed genes at total and individual stages in ‘Sungold’ and ‘F43’, respectively. (**b**) The proportions of genes expressed at four different expression levels in ‘Sungold’ (AO, AF1-AF8) and ‘F43’ (SO, SF1-SF8). (**c**) Pearson correlation coefficient (PCC) analysis of gene expression data from nine representative stages in ‘Sungold’ and ‘F43’. (**d**) Principal component analysis (PCA) plot showing clustering of transcriptomes of nine representative stages in ‘Sungold’ and ‘F43’.

To understand the molecular basis underlying the differences in cell size and cell number between *P. armeniaca* and *P. sibirica* during their fruit development, we performed RNA sequencing (RNA-seq) at nine representative stages from each of ‘Sungold’ and ‘F43’, the same as the anatomical samples (Figure 1b–d). An average of ~6.41Gb of clean data per sample was generated for a total of 54 samples (three independent biological replicates at each stage from two cultivars), and overall, about 89.99% of clean reads were mapped to the reference genome on average (Table S1). A total of 28,803 genes were detected to be expressed in at least one of the 54 samples, including 27,080 known and 1723 novel genes. The high Pearson correlation coefficient (PCC) between the expression values of the three biological replicates indicated the high quality of the replicates in each tissue sample (Figure S2), which allowed us to calculate the average fragments per kilobase of transcript length per million mapped reads (FPKM) values of the three biological replicates as the expression level of genes in 18 tissue samples for further analysis. The expressed genes in individual samples of ‘Sungold’ and ‘F43’ accounted for roughly 73.71%-83.32% of the total expressed genes (Figure 2a), and their ratios in ‘F43’ were higher than in ‘Sungold’ at every stage except stage F3. The proportions of genes distributed to four expression levels were relatively similar in all stages between the two cultivars (Figure 2b), and approximately 14.45%–16.85% of genes showed very high expression levels (FPKM > 50) in different samples.

To investigate the overall difference in the transcriptome dynamics during fruit development between ‘Sungold’ and ‘F43’, principal component analysis (PCA) and hierarchical clustering based on PCC were performed using the 28,803 expressed genes (Figure 2c,d). Consistent with the grouping of anatomical data, CDP and CEP indicated that transcriptome profiles were clustered in two groups within individual cultivars (Figure 2c,d and Figure S2). Moreover, the PCC results showed a relatively higher correlation of adjacent or similar stages during the CDP within and across the cultivars (Figure 2c). However, the different stages of the CEP exhibited relatively looser clustering and a lower correlation between the two cultivars. We noticed that AF7 and AF8 of ‘Sungold’ exhibited a tight relationship but were radically far from SF5–SF8 of ‘F43’. In addition, the key stages AF4 and AF5 in the first cell expansion phase showed substantial differences with the second cell expansion phase (AF7 and AF8) in ‘Sungold’, but they showed a closer correlation with SF4 of ‘F43’. We suggested that there were different transcriptional mechanisms for the two cell expansion processes in ‘Sungold’, but there was a highly similar transcriptional program to ‘F43’ during the first cell expansion process. Taken together, the above results indicated that the distinction in the transcriptional level was concentrated in the CEP between ‘Sungold’ and ‘F43’, which was consistent with the differences in cell growth characteristics throughout fruit development. Further, the particularity in the transcriptional level at stages AF7 and AF8 may be responsible for the larger fruits of *P. armeniaca*.

### 2.3. Identification of DEGs in the Cell Division and Cell Expansion Period between the Two Apricots

**Figure 3:**
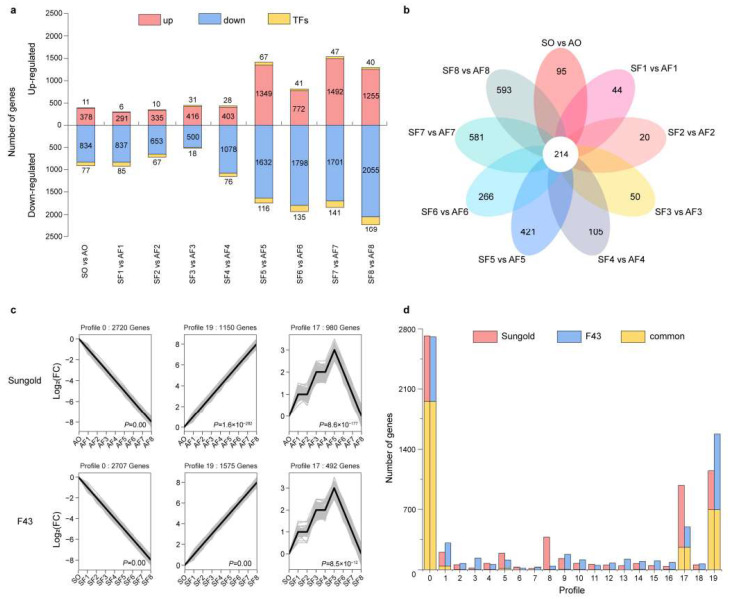
The numbers of DEGs and difference in expression patterns between ‘Sungold’ and ‘F43’ during fruit development. (**a**) The numbers of upregulated (upper red bars) and downregulated (lower blue bars) genes at each stage of fruit development in ‘Sungold’ as compared with ‘F43’. Yellow bars indicate the numbers of transcription factor (TF)-encoding genes. (**b**) The numbers of unique and common DEGs at different development stages. (**c**) The significant clustering profiles of the expression trends of all DEGs in ‘Sungold’ and ‘F43’ found using STEM analysis. The *x*-axis represents nine representative development stages, and the *y*-axis shows the log_2_ Fold-change (FC). (**d**) The numbers of unique and common DEGs in different STEM profiles between ‘Sungold’ and ‘F43’.

To investigate the details of transcriptional differences in the CDP and CEP, we identified the significant differentially expressed genes (DEGs) between ‘Sungold’ and ‘F43’ at each developmental stage. On the whole, 297-2244 DEGs (including 6-169 TF-encoding genes) exhibited significant (fold change > 2 and q-value < 0.05) upregulation or downregulation at nine comparisons of the corresponding stage in ‘Sungold’ as compared with ‘F43’ (Figure 3a). As we had expected, the numbers of DEGs at every stage of the CEP were higher than in the CDP, and so were the numbers of TF-encoding genes. The largest number of DEGs was found in the SF8 vs. AF8 comparison with 3519 genes followed by the SF7 vs. AF7 comparison (3381), and the lowest number of genes (965) was differentially expressed in the SF3 vs. AF3 comparison. Out of a total of 6480 significant DEGs, only 214 genes were common genes that were differentially expressed in all nine comparisons, and more unique DEGs were identified in the CEP than in the CDP (Figure 3b). In short, this set of DEGs with a high spatial–temporal resolution may be related to the larger fruits in ‘Sungold’.

To further identify the differences in the expression pattern between the two apricots throughout fruit development, we separately performed the short time-series expression miner (STEM) analysis for ‘Sungold’ and ‘F43’ by using all 6480 significant DEGs. The results showed that the DEGs in both cultivars were clustered into 20 predominant profiles (profiles 0–19), covering 6359 and 6402 significant DEGs in ‘Sungold’ and ‘F43’, respectively (Figure S3). Overall, these profiles showed divergent expression patterns within the cultivar but similar patterns between the two cultivars, reflecting the complexity and similarities of the molecular basis of fruit growth and development in apricots. Among these profiles, the DEGs were the most significant and most numerous clustered in profile 0 in both cultivars followed by profiles 19 and 17 (Figure 3c). The DEGs of profile 0 exhibited a decreasing trend during fruit development, which may be related to cell division and fruit set. However, profile 19 showed an increasing trend opposite to profile 0, the DEGs of which may be associated with cell expansion and fruit ripening (Figure 3c). In addition, the expression of genes reached its peak at stage F5 in profile 17 for both cultivars, indicating that these DEGs were highly expressed during the first cell expansion phase (phase II) in two cultivars (Figure 2e and Figure 3c). Through further comparative analysis, the number of DEGs varied in each similar profile (Figure 3d). The results suggested that the common genes with significantly different expression levels or unique genes at each expression pattern between the two cultivars may contribute to the difference in fruit size, especially in profiles 0, 19, and 17.

### 2.4. Identification of Underlying Genes and Regulators Related to Cell Division and Cell Expansion

**Figure 4:**
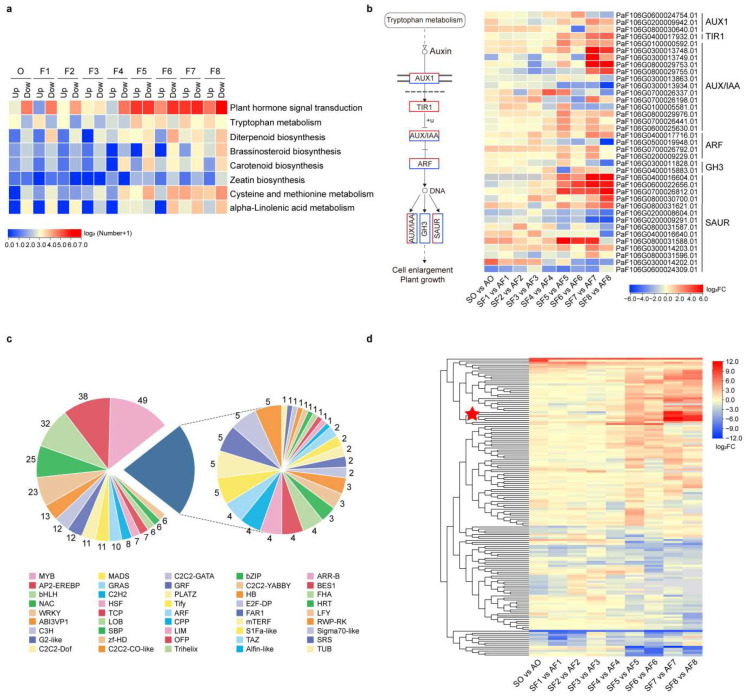
Characterization of underlying genes and regulators related to cell expansion and cell division. (**a**) The heat map shows the numbers of upregulated and downregulated genes related to the plant hormone at different stages during fruit development in ‘Sungold’ as compared with ‘F43’ based on log_2_ (Number + 1) values. (**b**) Schematic diagram and heat map of differences in expressions of DEGs involved in auxin signaling pathway during fruit development. Key enzymes expressed differently have colored rectangles (on the left) and genes linked to these enzymes as well as their log_2_ FC values presented in the heat map (on the right). The redder color represents a higher log_2_ FC, and the bluer color represents a lower log_2_ FC. The yellow color represents the median value, and the gray color represents not available (NA). AUX1, auxin influx carrier; TIR1, transport inhibitor response 1; AUX/IAA, auxin-responsive protein IAA; ARF, auxin response factor; GH3, auxin-responsive GH3 gene family; SAUR, SAUR family protein. (**c**) The number of differentially expressed TF genes between the two cultivars. (**d**) Heat map and clustering dendrogram of differences in expressions of DEGs related to cell expansion and cell division. A redder color represents higher log_2_ FC, and a bluer color represents lower log_2_ FC. The yellow color represents the median value, and the gray color represents NA. The red star indicates the branch with the most significant differences in gene expressions.

To screen for underlying genes and regulators involved in cell division or expansion, we preferentially dissected the functional annotation features of DEGs in the abovementioned six clustering gene profiles (Figure 3c). Considering the important roles of the phytohormones in fruit development and fruit size, we first identified a series of DEGs involved in plant hormone signal transduction, biosynthesis, and metabolism based on the KEGG enrichment results (Figure 4a). We found that a larger number of DEGs were related to signal transduction than biosynthesis and metabolism, most of which were concentrated in the CEP (Figure 4a). Differences in gene expressions involved in the signaling of eight phytohormones included auxin, gibberellins (GAs), cytokinins (CKs), abscisic acid (ABA), ethylene (ETH), brassinosteroids (BRs), jasmonic acid (JA), and salicylic acid (SA) between the two apricots were carefully observed (Figures S4 and S5). Although the DEGs dominated by upregulation and downregulation were enriched in all eight phytohormone signaling pathways, differences in expressions of global genes related to auxin signaling were more significant in the CEP, especially 1 upregulated *TIR1* (PaF106G0400017932.01), 5 upregulated *AUX/IAAs* (PaF106G0100000592.0, PaF106G0300013748.01, PaF106G0300013749.01, PaF106G0800029753.01, and PaF106G0800029755.01), and 5 upregulated *SAURs* (PaF106G0400016604.01, PaF106G0600022656.01, PaF106G0700026812.01, PaF106G0800030700.01, and PaF106G0800031621.01) in ‘Sungold’ (Figure 4b). However, we also noticed that several genes were significantly downregulated during the CEP of ‘Sungold’, including one *AUX/IAA* (PaF106G0300013934.01), one *ARF* (PaF106G0500019948.01), one *GH3* (PaF106G0300011828.01), and two *SAURs* (PaF106G0200008604.01 and PaF106G0200009291.01). Similar to the auxin signaling pathway, several key genes related to auxin biosynthesis in the tryptophan metabolism pathway were also highly expressed in the CEP of ‘Sungold’ compared to that of ‘F43’ (Figure S6), including one *TAA1* (PaF106G0800032361.01), two *AMIs* (PaF106G0300013477.01 and PaF106G0800030658.01), and five *DDCs* (PaF106G0600022121.01, PaF106G0600022118.01, PaF106G0700028546.01, PaF106G0200009721.01, and BGI_novel_G001118).

Apart from auxin, all the DEGs with the most significant differences in the signaling of each phytohormone showed downregulation during the CEP of ‘Sungold’, and there was PaF106G0100004578.01 (*B-ARR*) in CKs signaling, PaF106G0100003110.01(PYR/PYL) in ABA signaling, PaF106G0200007354.01 (*GID1*) in GAs signaling, PaF106G0100003338.01 (*EIN3*) in ETH signaling, PaF106G0500020009.01 (*BRI1*) in BRs signaling, PaF106G0700026668.01 (*MYC2*) in JA signaling, and PaF106G0800030743.01 (*PR-1*) in SA signaling (Figure S5). In addition, PaF106G0200009895.01 (*UGT76C*) related to CKs metabolism, PaF106G0600022029.01 (*GA2ox*) related to GAs biosynthesis, PaF106G0600023073.01 (*ABA2*) related to ABA biosynthesis, PaF106G0300014030.01 (*CYP90D1*) related to BRs biosynthesis, PaF106G0500019751.01 (*ACS*) related to ETH biosynthesis, and PaF106G0100002135.01 (*DAD*) related to JA biosynthesis as the DEG with the most significant differences in their respective pathways were also downregulated in ‘Sungold’ (Figure S6). In contrast, there were several DEGs in different hormone pathways with potentially positive components to the cell expansion of ‘Sungold’ interesting us, including one BRs biosynthesis gene *CYP90A1* (PaF106G0200009958.01), one ETH biosynthesis gene *ACO* (PaF106G0300012099.01), one ABA biosynthesis gene *NCED* (PaF106G0400017352.01), one ETH signaling gene *EBF* (PaF106G0800029757.01) and JA signaling gene *MYC2* (PaF106G0100006225.01), etc. (Figures S5 and S6). However, differences in overall gene expressions involved in the signaling of eight phytohormones, biosynthesis, and metabolism during the CDP between the two apricots were relatively not remarkable. In summary, a detailed analysis of differential expression in eight classical hormone-related genes suggested that auxin signaling took the wider and more important responsibility for cell size and fruit size than other hormones.

TFs play a vital role in the regulation of plant growth and development both temporally and spatially. We identified a total of 350 TFs corresponding to 45 families among the foregoing six clustering gene profiles (Figure 4c). The top 10 TF families in terms of the number were *MYB* (49), *AP2-EREBP* (38), *bHLH* (32), *NAC* (25), *WRKY* (23), *ABI3VP1* (13), *C3H* (12), *G2-like* (12), *C2C2-Dof* (11), and *MADS* (11). To distinguish the potential effects of TF families on cell expansion and cell division, we observed the distribution of differentially expressed TF families in six predominant clustering gene profiles from ‘Sungold’ and ‘F43’ (Figure S7). In total, the maldistribution of the number of TF-coding genes in different profiles within and between the two apricots implied that distinct TF families or members had a preference for the transcriptional regulation of cell division or cell expansion. For example, more TF-coding genes were distributed in profile 0 for most TF families in both apricots, which revealed that these TF-coding genes highly expressed during the CDP may play transcriptional regulatory roles in the cell division process. Nevertheless, we noticed that several *WRKYs*, *AP2-EREBPs*, *bHLHs*, *C3Hs*, and *GRASs* were distributed in profiles 19 and 17. Moreover, these TF genes were mainly highly expressed in the first or second half of the CEP and may play essential roles in transcriptional control for the cell expansion process.

Cell cycle machinery and cell wall loosening mechanisms drive the occurrence and development of cell division and cell expansion. Thus, we first picked out a series of DEGs that were associated with cell cycle control and cell wall loosening from six clustering gene profiles. For instance, the cell-cycle-related genes included *cyclin*, *cyclin-dependent kinase* and related proteins (*CDK*, *SMR*, and *ICK*), the *anaphase-promoting complex* (*APC*), and *cell division cycle* and *cell division control protein* (*CDC*) (Figure S8a); the cell-wall-related genes included *expansin* (*EXP*), *xyloglucan endotransglucosylase/hydrolases* (*XTH*), *xyloglucan endotransglycosylase* (*XET*), *cellulose synthase* and related proteins (*CESA*, *CSL*, and *CSI*), *endoglucanase* (*CEL*), and *pectinesterase* (*PME*) (Figure S8c). Second, we also detected a series of candidate genes that encode proteins homologous to major known QTLs for fruit-size-related traits, such as *CSR* (cell size regulator/FANTASTIC FOUR), *CYP78A* (cytochrome P450 subfamily), *TRM* (TONNEAU1 Recruiting Motif), *WUS* (WUSCHEL), *CLV3* (CLAVATA3), and *SUN* (IQ-domain) (Figure S8b). Third, DEGs involved in signaling pathways that determine organ size were identified as well, including the ubiquitin–proteasome pathway, the mitogen-activated protein kinase signaling pathway, G-protein signaling, and the IKU pathway (Figure S9). We observed and integrated differences in gene expressions of the above genes potentially related to cell division and cell expansion between the two apricots based on the log_2_ FC. The clustering results showed that the branch with the most significant differences in gene expressions had five genes consisting of three *EXPs* and two *CELs* (Figure 4d and Figure S8c), which were all highly expressed at stages AF7 and AF8 of the CEP of ‘Sungold’. Likewise, several DEGs from homologous genes of QTLs covering *CYP78A*, *CSR*, *TRM*, and *SUN* were highly expressed at stages AF7 and AF8 (Figure S8). Nevertheless, differences in expressions during the CDP between the two apricots for genes from the three categories described above were relatively insignificant.

**Figure 5:**
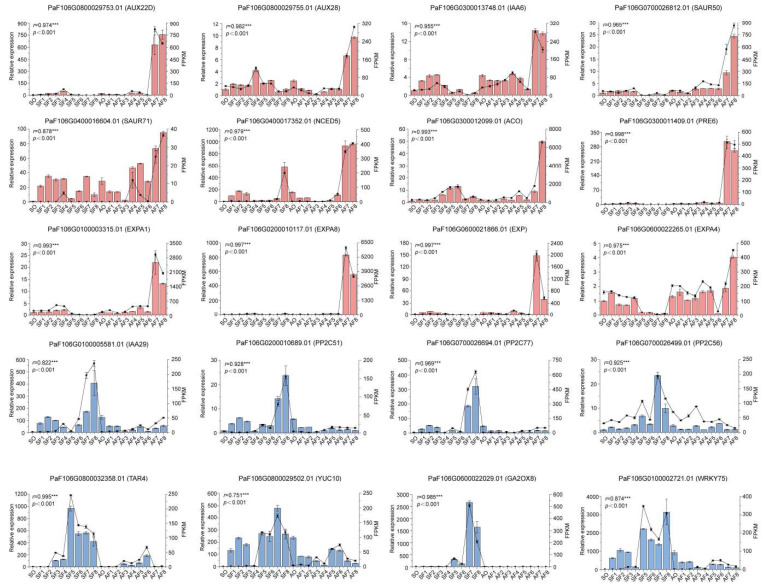
Expression levels of 20 candidate DEGs validated by qRT-PCR. Columns and lines indicate the mean values of expression levels of qRT-PCR and RNA-seq of the candidate DEGs, respectively. Error bars stand for the standard deviation of three replicates. Pearson correlation coefficients (*r*) were calculated between qRT-PCR and RNA-seq data of candidate DEGs. *** indicates a highly significant correlation (*p* < 0.001). Red color represents DEGs dominated by upregulation in ‘Sungold’, and blue color represents DEGs dominated by downregulation in ‘Sungold’.

To sum up, we took a closer look to find out the underlying genes involved in cell division and cell expansion at the gene set composed of six clustering gene profiles. The results suggested that multiple functional genes and regulators had transcriptional regulatory effects on the cell expansion process, which may result in larger cells and fruits in *P. armeniaca*. Moreover, we tested and analyzed the expression levels of 20 DEGs that were related to cell expansion in all 18 samples from ‘Sungold’ and ‘F43’ by using quantitative reverse transcription polymerase chain reaction (qRT-PCR) (Figure 5). The correlation coefficient between the RNA-seq and qRT-PCR was ≥0.80 (*p* < 0.001) for most of the tested genes (19/20), indicating the reliability of RNA-seq data to reflect the abundance of transcript levels.

### 2.5. Co-Expression Networks of Genes Associated with Cell Size

**Figure 6:**
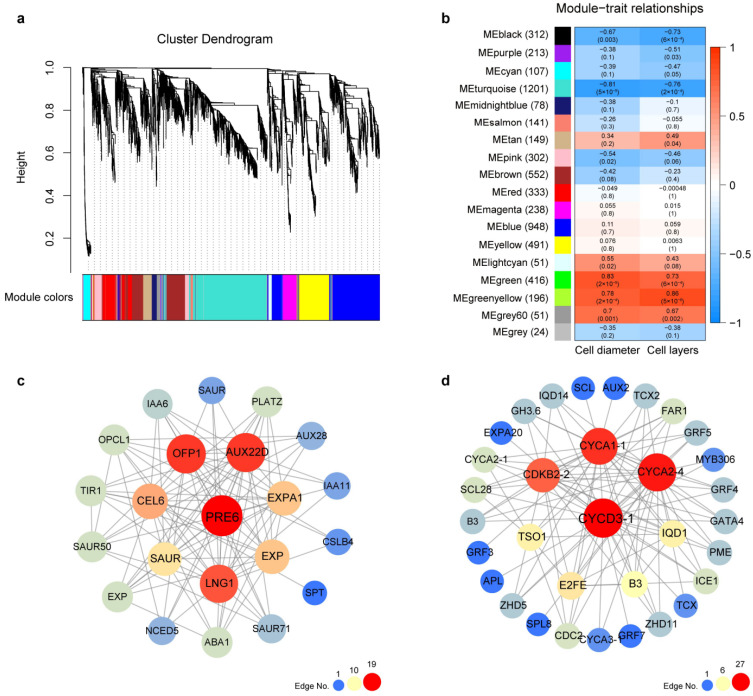
Identification of hub genes correlation with cell layers and cell diameter in the co-expression network. (**a**) Hierarchical clustering dendrogram presenting eighteen co-expression gene modules. Each leaflet in the tree corresponds to an individual gene. (**b**) Module–trait relationships based on Pearson correlations. The color from blue to red represents correlation coefficient (*r*) values from −1 to 1. (**c**) The co-expression network contained 22 co-expression genes for the MEgreen module. (**d**) The co-expression network contained 33 co-expression genes for the MEturquoise module.

To explore the relationship between the genes related to cell division and cell expansion mentioned above, we performed weighted gene co-expression network analysis (WGCNA) by using all the DEGs from the six clustering gene profiles. This gene set was divided into different eighteen co-expression modules (Figure 6a). Among them, MEgreen and MEyellowgreen modules showed significant positive correlations with the cell diameter and cell layers of the mesocarp, and MEturquoise and MEblack modules were significantly negatively correlated with cell diameter and cell layers (Figure 6b). 

Specifically speaking, the results showed that the MEgreen module containing 416 genes showed a significant positive correlation with cell diameter, with the highest correlation coefficient (*r*) of 0.83, and cell layers, with the second-highest correlation coefficient of 0.73. We picked out the underlying candidate genes in the MEgreen module based on the high module membership (MM) and high gene significance (GS), including the 14 phytohormone-related genes, 4 TF genes, and 6 structural genes or other regulators. However, based on the weight values (top 100) and degree of connectivity between candidate genes, only 22 genes were screened to construct a gene co-expression network. *PRE6/bHLH* (PaF106G0300011409.01) was identified as a key hub gene with 19 connected edges (Figure 6c). Significantly, *PRE6* mainly interacted with several auxin signaling genes (*TIR1*, PaF106G0400017932.01; *AUXs*, PaF106G0300013748.01, PaF106G0800029753.01, and PaF106G0800029755.01; *SAURs*, PaF106G0700026812.01, PaF106G0800031621.01, PaF106G0800030700.01, and PaF106G0400016604.01) and cell wall loosening related genes (*EXPs*, PaF106G0200010117.01, PaF106G0100003315.01, and PaF106G0600021866.01; *CEL6*, PaF106G0500019886.01). Considering the gene expression levels, we noticed that all 13 genes exhibited significantly higher expression at stages AF7 and AF8 of ‘Sungold’ as compared with ‘F43’ (SF7 and SF8) (Figure S11a) but no significant expression differences in the CDP. Therefore, these 13 genes were considered to be key candidate genes related to the positive regulation of cell size and fruit size in *P. armeniaca*.

In addition, we constructed gene co-expression networks for the other three significantly relevant modules according to the screening methods similar to the MEgreen module (Figure 6d and Figure S10). For the MEturquoise module, 3 phytohormone-related genes, 19 TF genes, and 11 structural genes or other regulators were screened to construct a gene co-expression network (Figure 6d). We found that the *CYC3D-1* (PaF106G0100005031.01) had the maximum number of connected edges (27), followed by *CYCA2-4* (PaF106G0300013744.01) and *CYCA1-1* (PaF106G0100005700.01). However, the differences in the expression of these genes were relatively weak both in the CDP and CEP (Figure S11b), so it was impossible to tell whether they were responsible for the differences in cell number or cell size between *P. armeniaca* and *P. sibirica*. For the MEgreenyellow and MEblack modules, although several genes showed expression differences in the CDP or CEP of the two apricot cultivars, the differences existed in multiple consecutive stages or in more complex situations (Figure S11c,d). Therefore, a comprehensive analysis of WGCNA results revealed that the MEgreen module provided a major gene co-expression network and 13 key candidate genes most relevant to the larger size of mesocarp cells and fruits in apricots.

## 3. Discussion

Fruit size is one of the most important quality and yield traits in apricots. The dynamic changes in fruit development from the aspects of phenotypic and cellular levels are the essential basis for investigating the variation in apricot fruit size. Generally speaking, the growth and development patterns for different fleshy fruit types within the Rosaceae can be depicted as either a single-sigmoid curve represented by pear or a double-sigmoid curve represented by peach [49,50]. In addition, the fruits of different varieties within a species may also exhibit two distinct growth patterns, such as strawberries [51]. These results indicate that differences in fruit growth patterns were not correlated to the type of fruit or the length of the development period. Thus, the diversities in the development characteristics of the aforementioned fruits are also not directly relevant to their fruit size. In this study, we performed phenotypic studies on the entire fruit development of cultivated large-fruit *P. armeniaca* ‘Sungold’ and wild small-fruit *P. sibirica* ‘F43’. We found that the differences in growth patterns between the two apricots mainly were concentrated in the additional second enlargement process before ‘Sungold’ turned ripe (Phase IV), even though ‘F43’ had a longer developmental period. In previous studies, the dynamic curves of fruit weight during the fruit development of different cultivars of *P. armeniaca* are very similar, including two rapid growth periods and reaching the highest values near ripening [52,53,54,55,56,57,58]. For *P. sibirica*, the fruit growth curve only has one growth peak and reaches 75%–85% of the final fruit weight in the early development period [59]. In general, although the conclusions are slightly different as to whether the fruit sizes of different *P. armeniaca* are ultimately determined by the first or the second rapid growth period [52,53,54,55,56,57], the differences in fruit development between *P. armeniaca* and *P. sibirica* in our study are consistent with previous results. Many studies have described that cell number (cell division) and cell size (cell expansion) are the major influencing factors to determine the final fruit size. Among them, cell number is recognized as a more important contributor to fruit size than cell size in certain crops, such as pear [60], sweet cherry [61], and plum [62]. Although the fruit sizes of some of them are correlated with the cell number of the ovaries, others are related to the cell number after anthesis [35]. Additionally, it is the case that cell size is the primary factor to influence the final fruit size for blueberry [63] and loquat [64]. Our anatomical studies showed that the major contributor to the larger fruit size in ‘Sungold’ was larger cell size, especially its additional second cell expansion process occurring in phase IV. In addition, we observed that the difference in the number of cell layers between the two apricots existed before flowering (stage O), though the difference was relatively less marked. Although the difference in cell diameter between the two apricots started at 21 DAFB (stage F3), the difference was more pronounced after stage F6 (70 DAFB). The above studies suggest that the second cell expansion process before ripening is an important breeding target for apricot fruit size in the future.

The molecular mechanisms underlying cell division and cell expansion in apricot fruit are poorly understood. We used the RNA-seq method to detect the differences in transcriptome dynamics between ‘Sungold’ and ‘F43’ at nine representative stages whose characteristics of cell layers and cell diameter were known. Differences in global transcriptome data and gene expressions in detail were all concentrated in the CEP, particularly stages F7 and F8. STEM analysis and its similar K-means method have been used to distinguish the differences in expression patterns in many species, such as apricot [65] and loquat [34]. Here, STEM analysis helped us to classify the significant DEGs according to their expression patterns in ‘Sungold’ and ‘F43’ separately. We obtained the gene set most likely associated with cell division and cell expansion from six primary clustering gene profiles (profiles 0, 19, and 17 for ‘Sungold’ and ‘F43’). In such an analysis, the unselected genes with more ups and downs in the remaining profiles may have pleiotropy at their respective different developmental stages for both kinds of apricots.

Phytohormones have been reported to affect organ size by altering the cell number or cell size [9]. For instance, a new study finds that the expression of an auxin signaling gene *MdAux/IAA2* is repressed, leading to an increase in cell size and fruit weight of apple [66]. This finding is inconsistent with our results, which show that the expressions of five *AUX/IAAs* (PaF106G0100000592.0, PaF106G0300013748.01, PaF106G0300013749.01, PaF106G0800029753.01, and PaF106G0800029755.01) are significantly upregulated during the second cell expansion process (stages AF7 and AF8) in our larger-fruit ‘Sungold’ compared to small-fruit ‘F43’ (Figure 4b). The research on *EuSAUR62* promoting cell elongation and thereby seed size is elucidated in Euryale [67], which supports our point that *SUARs* are positively related to the cell expansion during the CEP of ‘Sungold’, such as PaF106G0400016604.01, PaF106G0600022656.01, PaF106G0700026812.01, PaF106G0800030700.01, and PaF106G0800031621.01 (Figure 4b). In contrast, we also recognized that one *AUX/IAA* (PaF106G0300013934.01) and two *SAURs* (PaF106G0200008604.01 and PaF106G0200009291.01) were significantly downregulated during the CEP of ‘Sungold’, which could be candidate genes that promote the cell expansion after being suppressed in larger fruit. A *TIR1* (PaF106G0400017932.01) was highly expressed in the CEP of ‘Sungold’, which was reported to be involved in the auxin-mediated signaling core pathway during plant growth and development as an auxin receptor, regulating cell division and cell expansion [68]. Moreover, *ARFs* and *GH3s* have been repeatedly reported to negatively regulate cell division and cell elongation during organ growth, respectively [69,70,71,72]. In our study, one downregulated *ARF* (PaF106G0500019948.01) and one downregulated *GH3* (PaF106G0300011828.01) in the CEP of ‘Sungold’ caught our attention, which may inhibit cell expansion in small-fruit ‘F43’. Research shows that *AUX1* genes play an auxin transport role in regulating the key plant development processes [73], and yet differences in expressions of our three *AUX1* genes between the two apricots were not obviously associated with their differences in cell number or cell size. The highest differences in transcript abundance of each auxin signaling gene between the two apricots with divergent fruit sizes almost occurred during the CEP, and the situation was the same for most auxin biosynthesis genes, indicating that auxin pathways played more important roles in cell size than cell number for larger fruit in *P. armeniaca*.

In addition to auxin, the roles of other phytohormones and their interaction also have been reported in regulating organ development and organ size for years. For example, *EjBZR1* represses cell expansion by targeting BRs biosynthesis gene *EjCYP90A* and thereby affects fruit size in loquat [64]. Rapeseed silique length variations are also mediated by another BRs biosynthesis gene *BnaC7.ROT3*(*CYP90C1*), which affects cell elongation [74]. Moreover, the dysfunction of an ethylene biosynthesis gene *ACS2* inhibits cell division and leads to shorter fruit, suggesting that ethylene is the requirement for cucumber fruit elongation [75]. In another previous study, *RhGAI1*, encoding a *DELLA* protein, repressed the cell-expansion-related gene *RhCesA2* due to the inducement by *RhEIN3-3*, resulting in the suppression of rose petal cell expansion [76]. In tomato, ABA-deficient *notabilis/flacca* (*not/flc*) shows a reduced fruit size owing to smaller cell size [77]. According to the report of Park et al., *CYTOKININ-RESPONSIVE GROWTH REGULATOR* (*CKG*) increases cell size and promotes the progression of the cell cycle in *Arabidopsis* [78]. In addition, the genes in the JA signaling pathway are recognized as negative regulators to repress seed size by regulating integument cell proliferation in *Arabidopsis*, such as *COI1*, *MYC2*, and *JAR1* [79]. SA usually acts to attenuate plant growth by interacting with several aspects related to auxin, including biosynthesis, metabolism, transport, signaling, and response [80]. In the current study, the differences in expressions of DEGs enriched in seven phytohormones’ (except for auxin) biosynthesis, metabolism, and signaling pathways were more significant in the CEP between the two apricots and dominant by downregulating in ‘Sungold’. It is worth mentioning that an upregulated BRs biosynthesis gene PaF106G0200009958.01 *(CYP90A1*) in ‘Sungold’ probably performs a similar function in promoting cell expansion with *EjCYP90A*, thus increasing the fruit size [64]. One ET biosynthesis gene *ACO* (PaF106G0300012099.01) was highly expressed in the CEP of ‘Sungold’, which was reported to be essential for carpel development in cucumber [75]. 

Transcription factors are essential for fruit growth and development [20]. However, the transcriptional regulatory network in the determination of fruit size remains to be explored. In our identified DEGs dataset, about 89.74% (350/390) TF-coding genes were distributed in the six primary clustering gene profiles, suggesting a differential transcriptional control in cell division and cell expansion between the two apricots. Some of the known TF members from the *MYB* family [81,82], *AP2-ERF* family [83,84], *bHLH* family [85,86], *NAC* family [87,88], and *WRKY* family [89] are the key components of the regulatory network in organ size through regulating cell division and cell expansion, which are also the top five TF families that contain more DEGs in our six clustering gene profiles. Our dataset showed that the proportions of TF-coding genes distributed in profile 0 in ‘Sungold’ and ‘F43’ were 68.60% and 64.88%, respectively, which revealed that most of TFs were highly expressed in the CDP and less expressed in the CEP. That is to say, these TFs distributed in profile 0 may be positively associated with the rapid cell division process or negatively associated with the rapid cell expansion process. A few TF families, represented by *GRF*, *E2F-DP*, and *YABBY*, were only distributed in profile 0 and were reported to be involved in the regulation of the cell division process [90,91,92]. What is more, several *WRKYs*, *AP2-EREBPs*, *bHLHs*, *C3Hs*, and *GRASs* genes were highly expressed in the first half or second half of CEP and less expressed in the CDP (Figure S7). Previous research shows that cell elongation can be regulated by antagonism between *HLH/bHLHs* in *Arabidopsis* and rice [86]. *C3H15* is identified as a negative regulator to control cell elongation in *Arabidopsis* [93]. In tomato, silencing *GRAS2* can reduce fruit weight by modulating cell size [94]. Instead, overexpressing *AP2/ERF* transcription factor *BOL* leads to a decrease in organ size via the regulation of cell size and cell number in *Arabidopsis* [84]. Therefore, it was reliable that we searched positive TF genes related to cell expansion or negative TF genes related to cell division from clustering gene profiles 19 and 17.

To understand the mechanisms behind cell division and cell expansion in fruit, a set of genes that participated in these processes have been identified. Among them, the most mentioned structural genes and regulators included cell cycle progression and cell-division-related genes [95], cell wall loosening and cell-expansion-related genes [96,97,98], homologous genes with known QTLs for fruit-size-related traits [15], and genes involved in main signaling pathways consisting of the ubiquitin–proteasome pathway, the mitogen-activated protein kinase signaling pathway, G-protein signaling, and the IKU pathway [99]. Reports indicate that the ubiquitin–proteasome pathway, the mitogen-activated protein kinase signaling pathway, and G-protein signaling control organ size by influencing the cell proliferation process generally in different plant species [99], the same can be said for QTLs, namely *CYP78A* and *SUN* [2]. However, only a few QTLs and their homologs are found to be responsible for altering cell expansion, including *TRM* and *CSR* [2,100]. By comparing and clustering the expression differences of the above candidate genes related to cell division and cell expansion between the two apricots based on the log_2_ FC, we found that several genes were significantly highly expressed at stages AF7 and AF8 of ‘Sungold’, belonging to various gene families including *CYP78A*, *CSR*, *TRM*, *SUN*, *EXP,* and *CEL* individually (Figure S8). Hence, the effects of these genes on cell division and cell expansion for apricot are not exactly the same as those previously studied in various plant species. For two other popular QTLs, namely *WUS* and *CLV3*, which were recognized as classical signaling pathways to regulate tomato fruit size by increasing the numbers of locules [2], we also searched their homologs and did not understand their effects on apricot fruit size. As for the well-known major QTL named *FW2.2/CNR* for controlling cell division in tomato [32], we merely found a few of the genes encoding PLAC8-containing proteins that participate in cadmium resistance or Ca2^+^ signaling rather than cell number regulation in six clustering gene profiles. Broadly speaking, differences in expressions of this gene set during the CDP between the two apricots were relatively insignificant, even for the cell-cycle- and cell-division-related genes, but most of these genes exhibited significant expression differences during the CEP, especially certain DEGs involved auxin signaling and cell wall loosening.

WGCNA provides a powerful and effective way to mine key candidate genes and significant gene co-expression networks for important traits of plants using expression data and phenotypic data. We used WGCNA to explore the relationship among a range of phytohormones-related genes, TF-encoding genes, structural genes, and other regulators that may be involved in the regulation of cell number and cell size. In our results, we observed that each module had a consistent correlation with the two kinds of phenotypic data, and the correlation coefficients for the same module were very close, which may be due to a strong correlation (*r* = 0.925**) between the cell diameter and cell layers in all 18 tissue samples from two apricots. To avoid missing important clues, we searched key candidate genes related to cell number and cell size in the four significantly correlated modules. Ultimately, we identified *PRE6/bHLH* as a hub gene, which interacted with 1 *TIR1*, 3 *AUX/IAAs*, 4 *SAURs*, 3 *EXPs*, and 1 *CEL*. These 13 genes were significantly highly expressed in stages AF7 and AF8 of ‘Sungold’ compared to ‘F43’, which were prioritized as key candidate genes for positively regulating cell size and fruit size in *P. armeniaca.* In *Arabidopsis*, *PRE6* negatively regulates auxin responses as a transcriptional repressor [101]. From this, it seems clear that our results are not consistent with *Arabidopsis* and that we need to further investigate the mechanisms of how *PRE6/bHLH* interacts with auxin signaling genes and cell wall loosening genes.

In our study, larger cell size was identified as a major contributor to larger fruit size in apricot, and several cell wall loosening related genes were identified as key candidates for controlling cell size. However, in addition to the mechanism of cell wall loosening, endopolyploidy by means of endoreduplication plays an important role in the determination of cell size [11]. In previous studies, endoreduplication was shown to be widespread and influence the organ size in multiple plants, such as *Arabidopsis* [102], tomato [11], maize [103], etc. We observed the expression difference in several genes reported to be involved in endoreduplication between the two apricots, such as *CDKs*, *CDCs*, *CYCs*, and *APCs* (Figure S8a), but it is difficult to correlate it with differences in expression because we lack observational data on cell ploidy. Moreover, the effect of cell ploidy on cell size has not been reported in apricot, which may be something we need to perfect in the future.

From a comprehensive perspective, our identification of the key fruit and cell growth period in large fruits can provide a valuable reference for the effective implementation of cultivation measures in apricots, making the cultivator supply apricots with water and nutrients at the optimal time that promotes the growth and development of fruits. Key candidate genes that regulate cell size and fruit size can serve as important targets for improving fruit size through gene function studies in the future. Furthermore, a relatively comprehensive comparison of the expression levels of genes involved in fruit size between *P. armeniaca* and *P. sibirica* yields more new insights into genetic improvement, molecular breeding, and evolutionary studies of apricot.

## 4. Materials and Methods

### 4.1. Plant Materials and Phenotyping

The fruit weight data of 95 cultivated *P. armeniaca* and 71 wild *P. sibirica* at maturity were collected (Figure 1a and Table S2). Among the 166 accessions, the fruit weight data for 28 accessions of apricot were quoted from *China fruit-plant monographs, apricot flora* [104]. Two apricot cultivars ‘Sungold’ (*P. armeniaca*) and ‘F43’ (*P. sibirica*) were grown at the long-term experiment base of the Research Institute of Non-timber Forestry in Mengzhou City, Henan Province, China. For both cultivars, we observed and randomly collected the ovaries from three biological replicates each day during the budding period to determine the full blooming day and the dynamics of ovary development. Then, we observed and randomly collected the developing fruits of each cultivar in triplicate from the full blooming day to the fruit ripening day once a week. Fifteen fresh fruits of each cultivar were randomly sampled on each harvest day and measured to determine the fruit weight by using an electronic analytical balance (AL 204, METTER TOLEDO, Shanghai, China) with 0.0001 g sensitivity. The ripe fruit of ‘Sungold’ and ‘F43’ were harvested 84 and 98 days after full blooming (DAFB), respectively.

Samples of nine representative developmental stages including the ovaries collected 5 days before full blooming (−5, O) and the fruits collected 0 (F1), 7 (F2), 21 (F3), 35 (F4), 49 (F5), 70 (F6), 77/91 (F7), and 84/98(F8) DAFB from ‘Sungold’ and ‘F43’ were reserved for further experiments: one part was snap-frozen in liquid nitrogen and then stored at −80 °C; the other part was fixed with FAA fixative (50% ethanol: glacial acetic acid: formaldehyde = 18:1:1) >24 h. To reduce the errors of sampling accuracy, we made different strategies for handling samples before they were frozen according to the development situation for both cultivars: samples at stages O, F1, and F2 were derived from whole ovaries or fruits; samples at stage F3 were derived from fruits after removing the kernels; and samples were only retained fleshy mesocarp after stage F4. All fixed samples were taken from intact ovaries or fruits.

### 4.2. Paraffin Section Preparation and Cell Measurement

The fixed samples from two cultivars at nine representative developmental stages adopted different precutting strategies based on the development situation before embedding paraffin: samples were cut at the equatorial position of the whole ovary or fruit before stage F4, whereas the samples after stage F5 were cut at the equatorial position of the mesocarp (including epicarp), as shown in Figure S12. All samples after pretreatment were subjected to dehydration by gradient alcohol application, embedded into the paraffin, and sliced using an RM2016 LEICA slicer (Leica, Wetzlar, German). Then, all the prepared sections were stained with saffron and solid green. Pictures of the paraffin sections were visualized using a Nikon optical microscope (Nikon Eclipse E100, Tokyo, Japan) and panoramic scanning software CaseViewer v2.4.0. The cell layers and cell diameter at the equatorial position of the mesocarp from the nine representative developmental stages for two cultivars were counted and measured by CaseViewer v2.4.0.

### 4.3. RNA Sequencing (RNA-Seq) Analysis

RNA-seq and bioinformatic analysis was conducted on the ovaries and fruits of ‘Sungold’ and ‘F43’ at nine representative developmental stages (BGI—Shenzhen, China). In brief, total RNAs from 18 tissue samples were purified by using an ethanol precipitation protocol and CTAB-PBIOZOL reagent according to the manual instructions (three biological replicates per sample), and then they were qualified and quantified using a Nano Drop and Agilent 2100 bioanalyzer (Thermo Fisher Scientific, Waltham, MA, USA). Library construction and sequences were generated on the DNBSEQ platform (BGI-Shenzhen, China). The clean data were filtered from the raw data by using SOAPnuke v1.5.6 and then aligned to the *P. sibirica* reference genome (cultivar ‘F106’, Genome Database for Rosaceae, tfGDR1049) by using HISAT2 v2.0.4 [105] and Bowtie2 v2.2.5 [106]. Novel transcript sequences were assembled using StringTie (http://ccb.jhu.edu/software/stringtie (accessed on 10 February 2022)).

### 4.4. Transcriptome Differential Analysis

Gene expression was calculated based on the fragments per kb per million fragments (FPKM) by using RSEM v1.2.12 [107]. A total of 28,803 genes expressed (FPKM > 0) in at least 1 of the 54 samples were used to analyze the transcriptome differences within and between the two apricots. Pearson correlation coefficients were analyzed, and the heat maps with hierarchical clustering were plotted using TBtools v1.098745 [108]. The principal component analysis was conducted on the OmicShare tools platform, a free online platform for data analysis (https://www.omicshare.com/tools/ (accessed on 13 May 2022)).

### 4.5. Differentially Expressed Genes (DEGs) Analysis

DEGs were identified across nine pairwise comparisons of SO vs. AO, SF1 vs. AF1, SF2 vs. AF2, SF3 vs. AF3, SF4 vs. AF4, SF5 vs. AF5, SF6 vs. AF6, SF7 vs. AF7, and SF8 vs. AF8 by using DESeq2 [109]. The significant DEGs at each comparison were screened with |log_2_FC| > 2 and a q-value < 0.05. To reduce the transcription noise, a total of 6480 significant DEGs with average FPKM values > 10 in at least 1 of the 18 tissue samples were chosen to perform further analysis. For the two apricot cultivars, gene expression pattern clustering analysis was performed separately with default parameters by using short time-series expression miner software (STEM) [110] on the OmicShare tools platform (https://www.omicshare.com/tools/Home/Soft/trend (accessed on 17 May 2022)). The Kyoto Encyclopedia of Genes and Genomes (KEGG) pathway enrichment analyses of DEGs were performed, and a q-value ≤ 0.05 was considered as the criterion for significant enrichment. The transcription factors (TFs) were identified by using PlantTFDB (http://planttfdb.gao-lab.org/ (accessed on 18 February 2022)). Functional annotations of DEGs were conducted using BLAST against the NCBI NR database (https://blast.ncbi.nlm.nih.gov/Blast.cgi (accessed on 21 February 2022)). The differences in gene expressions in the 9 comparisons and gene expressions in 18 samples were visualized by using TBtools [108] based on log_2_FC and log_2_ (FPKM + 1), respectively.

### 4.6. Quantitative Reverse Transcription Polymerase Chain Reaction (qRT-PCR) Validation

Total RNA from each tissue sample (18 tissue samples in total) used for qRT-PCR analysis was obtained from a mixture of the three individual biological replicates at each development stage. All 18 RNA samples were reverse transcribed using Goldenstar RT6 cDNA Synthesis Mix, and the obtained cDNA was diluted 5-fold for qRT-PCR validation. Specific primers of 20 candidate genes designed using Primer-BLAST (https://www.ncbi.nlm.nih.gov/tools/primer-blast/ (accessed on 20 July 2022)) are listed in Table S3, and *UBC* (ubiquitin-conjugating enzyme) was used to normalize the transcript level as an internal control gene [111]. All qRT-PCR experiments were performed in at least three technical replicates, and the relative expression was calculated using the 2^−ΔΔCt^ method for each candidate gene in each sample. 

### 4.7. WGCNA and Visualization of Gene Networks

A total of 5803 significant DEGs from six major clustering profiles from two cultivars (profiles 0, 19, and 17) were used to perform weighted gene co-expression network analysis (WGCNA) by using the WGCNA in R [112]. The modules were obtained using default settings, but the soft power was 12. The minimum module size was 30, and the merge cut height was 0.25. The correlation-based associations between phenotypic data (mesocarp cell diameter and mesocarp cell layers) and gene modules were calculated using the default settings. To identify the relationship between the phytohormones-related genes, TF genes, key structural genes, and other regulators, the genes with the functional annotation described above within each significant module were selected based on high module membership (|MM| > 8, *p*-value < 0.05) and gene significance (|GS| > 5, *p*-value < 0.05). The major co-expression networks for each significant module based on the weight values (top 100) and degree of connectivity between the above-selected candidate genes were exported using Cytoscape v3.9.1.

### 4.8. Statistical Analysis and Plotting

In the analysis of the difference in fruit weight between *P. armeniaca* and *P. sibirica*, the significance boxplot with a *T*-test was plotted by using the OmicShare tools platform. In qRT-PCR analysis, the final expression data of qRT-PCR and RNA-seq of 20 candidate genes at each development stage were presented with a mean value and standard deviation (mean ± SD, n = 3). The Pearson correlation coefficients (*r*) and statistical significance (*p*) between RNA-seq and qRT-PCR were evaluated based on their respective mean value at each stage using SPSS v27.0.1.0 software. The 95% confidence intervals for the Pearson correlation coefficient of each tested gene are listed in Table S4. Unless otherwise noted, figures used to display the statistics were plotted by using OriginPro 2021 software.

## 5. Conclusions

Based on the comparative analysis of anatomical and transcriptomic dynamics during fruit growth and development in two apricot cultivars with contrasting fruit sizes, this study exhibited that the difference in fruit size was mainly caused by the difference in cell size between *P. armeniaca* and *P. sibirica*. We comprehensively analyzed the details in expression differences of DEGs between two apricot cultivars that may affect cell number or cell size, and these results suggested that the *PRE6/bHLH*, auxin signaling pathway, *expansins*, and their interaction with each other had the most important effect on the positive transcriptional regulation of cell size, resulting in the larger fruit in *P. armeniaca*. The results provide a valuable resource to dissect the role of key candidate genes controlling fruit development and fruit size in apricot. In addition, our findings provide evidence and inspiration that cell size is a promising objective for fruit size breeding in apricot in the future.

## Data Availability

The raw transcriptome sequencing reads were submitted to the Sequence Read Archive (https://www.ncbi.nlm.nih.gov/sra (accessed on 6 February 2023)) under accession number PRJNA931767.

## Supplementary Materials

The supporting information can be downloaded at: https://www.mdpi.com/article/10.3390/ijms24108748/s1.
