# Supplementary material for: Comparative Anatomical and Transcriptomics Reveal the Larger Cell Size as a Major Contributor to Larger Fruit Size in Apricot
Source: Int J Mol Sci. 2023 May 14;24(10):8748. doi: 10.3390/ijms24108748 (PMC10218707; doi:10.3390/ijms24108748)
Supplement: Supplementary file 1 [file ijms-24-08748-s001.zip › ijms-2351827-supplementary/Supplementary materials.pdf]

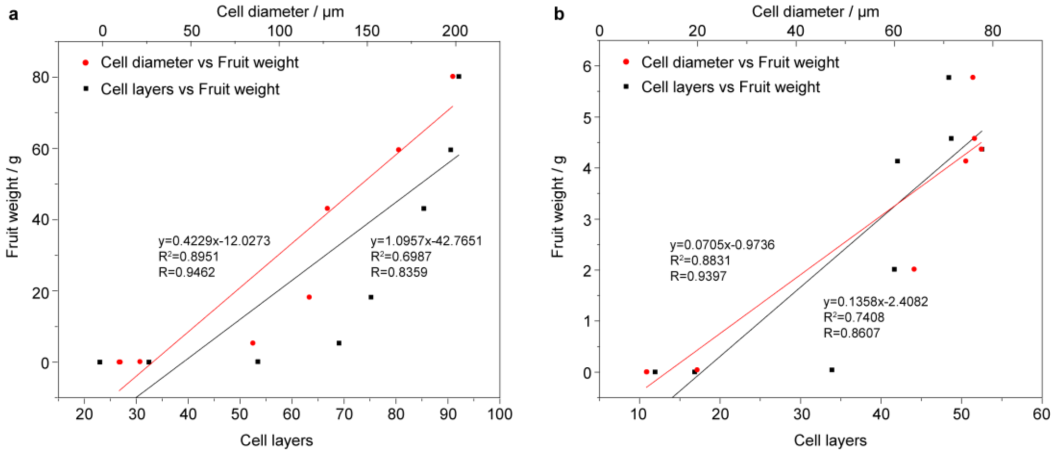

Figure S1. Relationships among fruit weight and fruit cell characteristics in the two apricots during development. a. Cell diameter is more strongly positively correlated with fruit weight in 'Sungold' than cell layers. **b**. Cell diameter is more strongly positively correlated with fruit weight in 'F43' than cell layers.

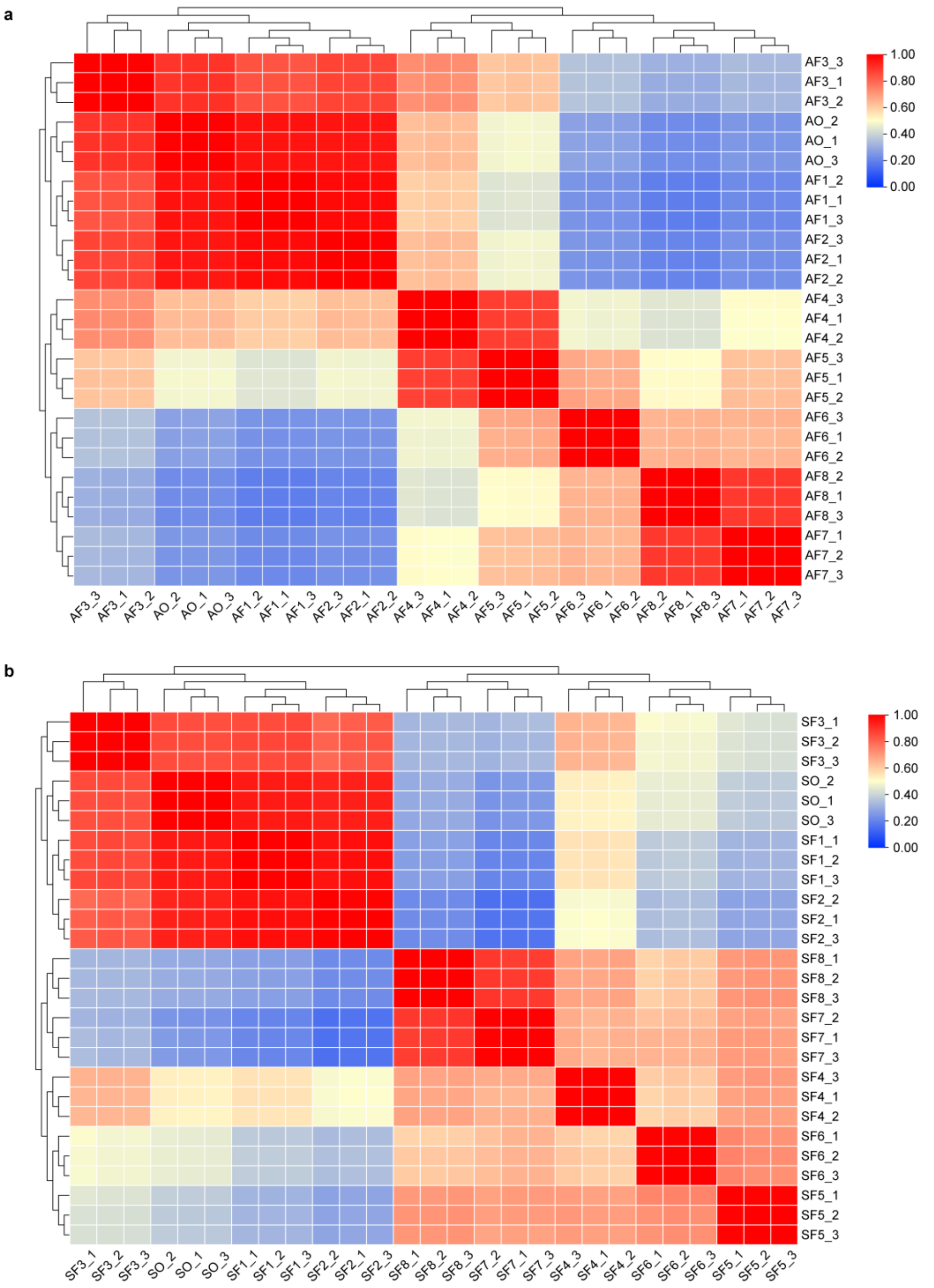

Figure S2. Heat maps showing the correlation between transcriptomes of three biological replicates of each tissue sample from 'Sungold' and 'F43'. a. Pearson correlation coefficient (PCC) among the three biological replicates of nine developmental stages in 'Sungold' (AO, AF1-AF8) is shown. **b**. PCC among the three biological replicates of nine developmental stages in 'F43' (SO, SF1-SF8) is shown. 1-3, three biological replicates of each sample.

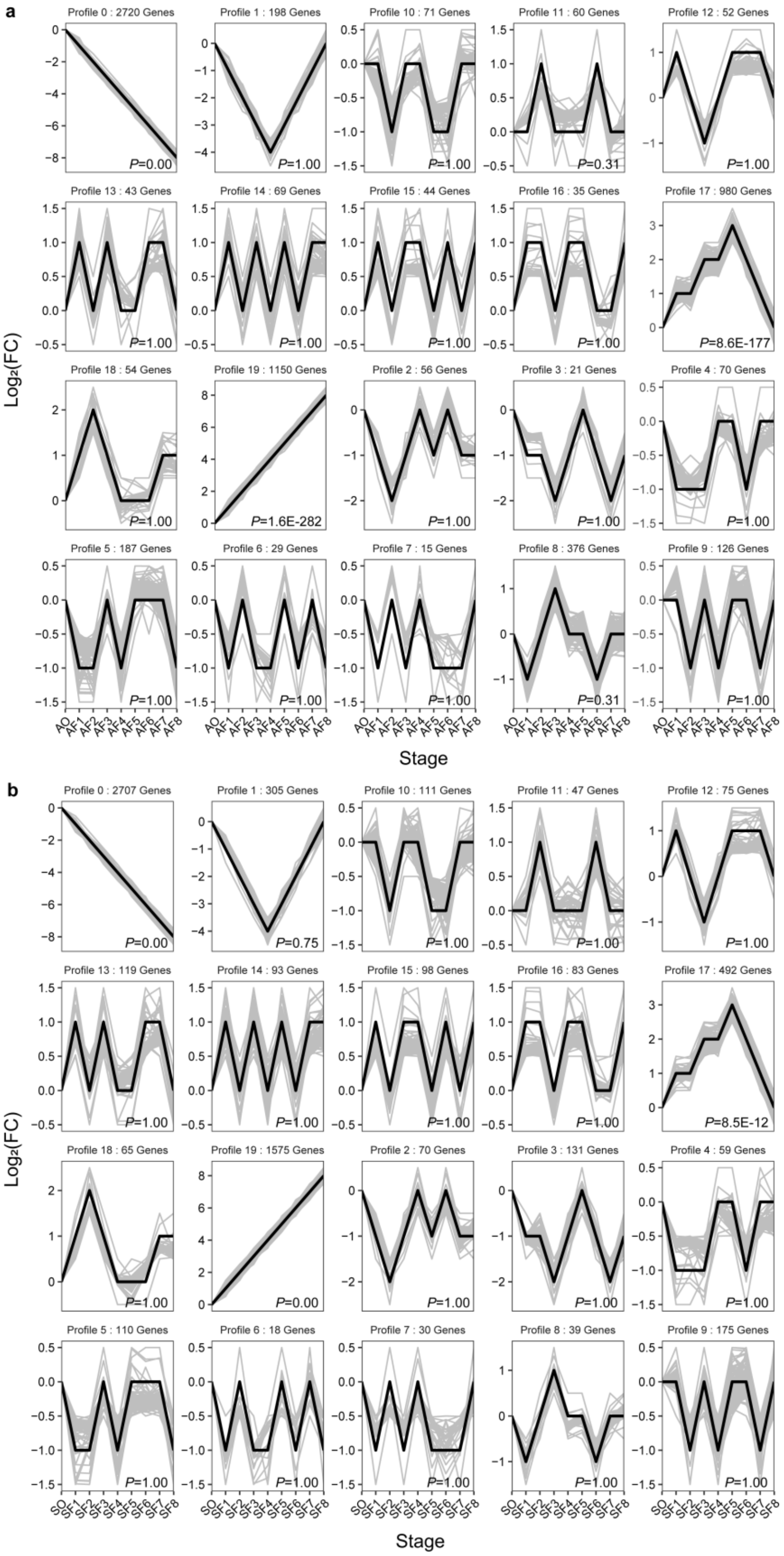

Figure S3. The clustering profiles of the expression trends of all DEGs using STEM analysis. The x-axis represents nine representative development stages and the y-axis shows the log_2Fold-change (FC). **a**.'Sungold'. **b**. 'F43'.

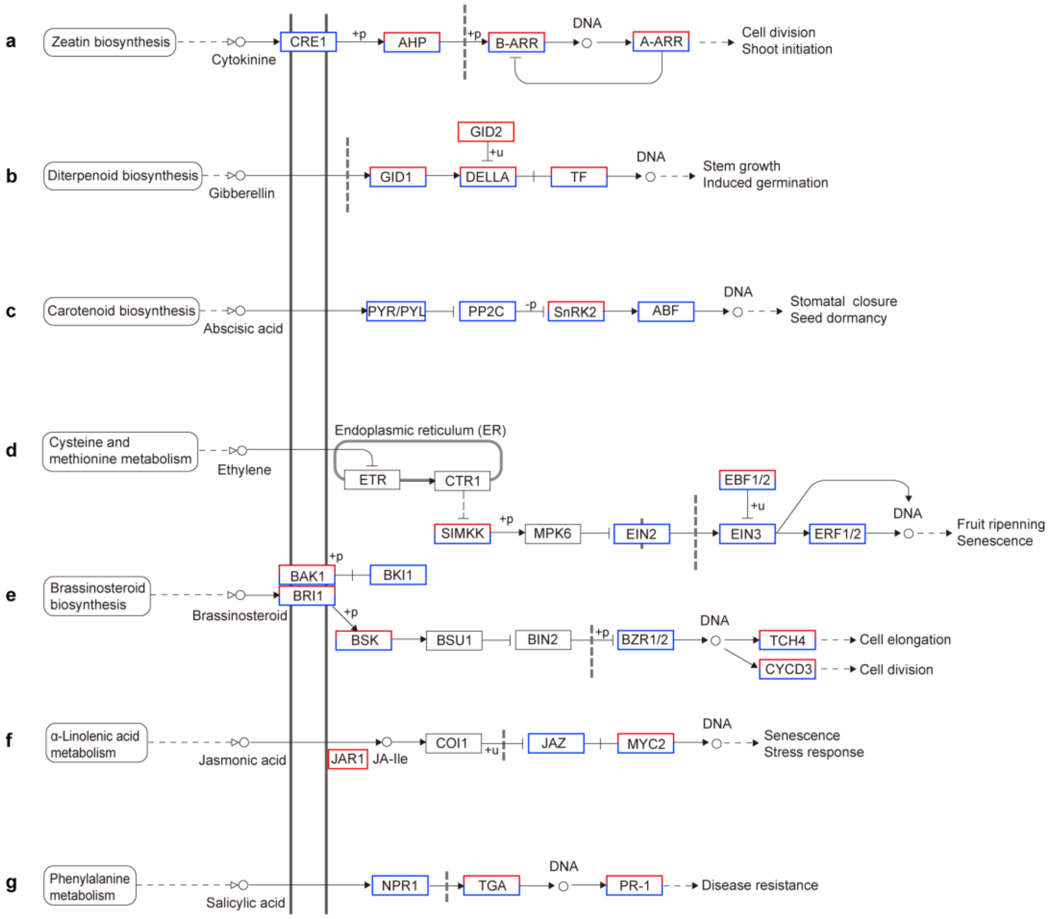

Figure S4. Schematic diagram of DEGs enriched in plant hormone signal transduction pathway in all nine comparisons between 'Sungold' and 'F43'. Key enzymes with DEGs enrichment have colored rectangles. The red indicates the DEGs dominated by upregulation, and the blue indicates DEGs dominated by downregulation. **a**. Cytokinine signaling pathway. **b**. Gibberellin signaling pathway. **c**. Abscisic acid signaling pathway. **d**. Ethylene signaling pathway. **e**. Brassinosteroid signaling pathway. **f**. Jasmonic acid signaling pathway. **g**. Salicylic acid signaling pathway.

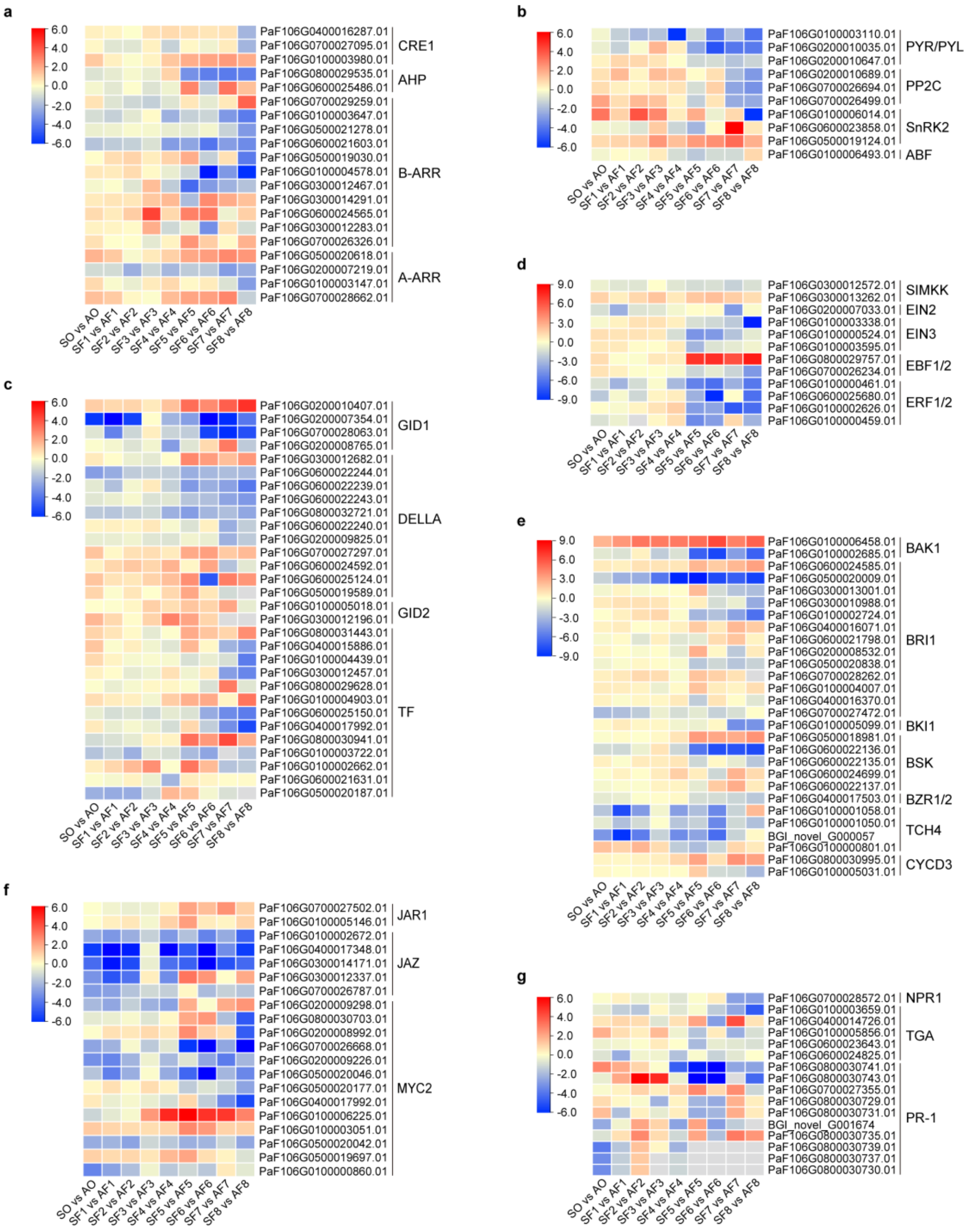

Figure S5. Heat maps showing the differences in expressions of DEGs link to key enzymes involved in plant hormone signal transduction. The redder color represents the higher log_2FC, and the bluer color represents the lower log_2FC. The yellow color represents the median value, and the gray color represents not available (NA). **a**. Cytokinine. CRE1, arabidopsis histidine kinase; AHP, histidine-containing phosphotransfer peotein; B-ARR, two-component response regulator ARR-B family; A-ARR, two-component response regulator ARR-A family; **b**. Abscisic acid. PYR/PYL, abscisic acid receptor PYR/PYL family;

PP2C, protein phosphatase 2C; SnRK2, serine/threonine-protein kinase SRK2; ABF, ABA responsive element binding factor; **c**. Gibberellin. GID1, gibberellin receptor GID1; DELLA, DELLA protein; GID2, F-box protein GID2; TF, phytochrome-interacting factor 4; **d**. Ethylene. SIMKK, mitogen-activated protein kinase kinase; EIN2, ethylene-insensitive protein 2; EIN3, ethylene-insensitive protein 3; EBF1/2, EIN3-binding F-box protein; ERF1/2, ethylene-responsive transcription factor; **e**. Brassinosteroid. BAK1, brassinosteroid insensitive 1-associated receptor kinase 1; BRI1, protein brassinosteroid insensitive 1; BKI1, BRI1 kinase inhibitor 1; BSK, BR-signaling kinase; BZR1/2, brassinosteroid resistant 1/2; TCH4, xyloglucan: xyloglucosyl transferase TCH4; CYCD3, cyclin D3; **f**. Jasmonic acid. JAR1, jasmonic acid-amino synthetase; JAZ, jasmonate ZIM domain-containing protein; MYC2, transcription factor MYC2; **g**. Salicylic acid. NPR1, regulatory protein NPR1; TGA, transcription factor TGA; PR-1, pathogenesis-related protein 1.

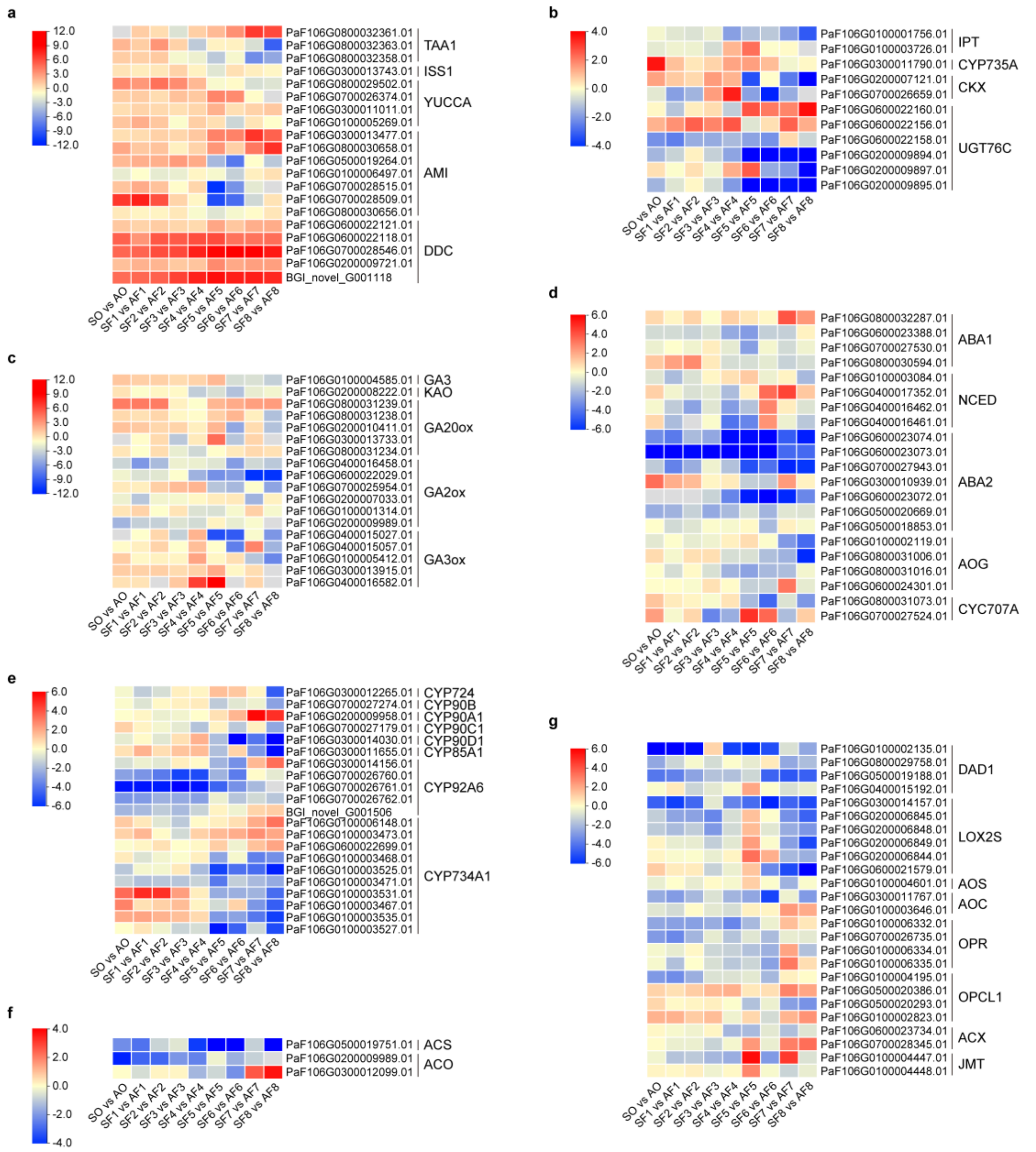

Figure S6. Heat maps showing the differences in expressions of the DEGs link to key enzymes involved in plant hormone biosynthesis and metabolism. The redder color represents the higher log_2FC, and the bluer color represents the lower log_2FC. The yellow color represents the median value, and the gray color represents NA. **a**. Auxin. TAA1, L-tryptophan-pyruvate aminotransferase; ISS1, aromatic aminotransferase; YUCCA, indole-3-pyruvate monooxygenase; AMI, amidase; DDC, aromatic-L-amino-acid/L-tryptophan decarboxylase; **b**. Cytokinins. IPT, adenylate dimethylallyltransferase; CYP735A, cytokinin trans-hydroxylase; CKX, cytokinin dehydrogenase; UGT76C, cytokinin-N-glucosyltransferase; **c**. Gibberellins. GA3, ent-kaurene oxidase; KAO, ent-kaurenoic acid monooxygenase; GA20ox, gibberellin-44 dioxygenase; GA2ox, gibberellin 2beta-dioxygenase; GA3ox, gibberellin 3beta-dioxygenase; **d**. Abscisic acid. ABA1, zeaxanthin epoxidase; NCED, 9-cis-epoxycarotenoid dioxygenase; ABA2, xanthoxin dehydrogenase; AOG, abscisate beta-glucosyltransferase; CYP707A, (+)-abscisic acid 8'-hydroxylase; **e**. Brassinosteroid. CYP724/CYP90B, steroid 22S-hydroxylase; CYP90A1, 3beta,22alpha-

dihydroxysteroid 3-dehydrogenase; CYP90C1/CYP90D1, 3-epi-6-deoxocathasterone 23-monooxygenase; CYP85A1, brassinosteroid 6-oxygenase; CYP92A6, typhasterol/6-deoxotyphasterol 2alpha-hydroxylase; CYP734A1, PHYB activation tagged suppressor 1; **f**. Ethylene. ACS, 1-aminocyclopropane-1-carboxylate synthase; ACO, aminocyclopropanecarboxylate oxidase; **g**. Jasmonic acid. DAD1, phospholipase A1; LOX2S, lipoxygenase; AOS, hydroperoxide dehydratase; AOC, allene oxide cyclase; OPR, 12-oxophytodienoic acid reductase; OPCL1, OPC-8:0 CoA ligase 1; ACX, acyl-CoA oxidase; JMT, jasmonate O-methyltransferase.

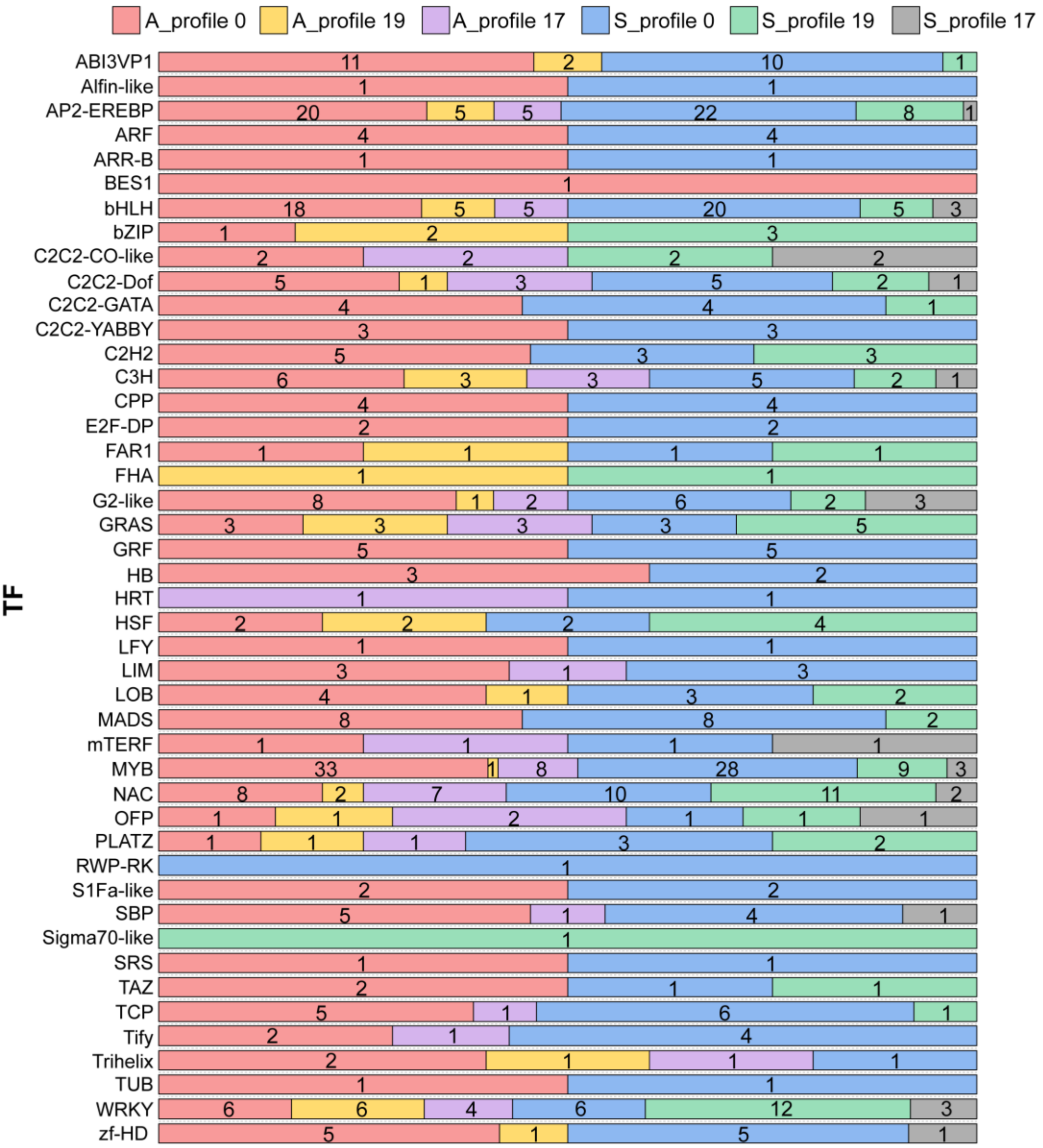

Figure S7. Distribution of differentially expressed TF families in six predominant clustering gene profiles from ‘Sungold’ and ‘F43’. A represents ‘Sungold’ and S represents ‘F43’.

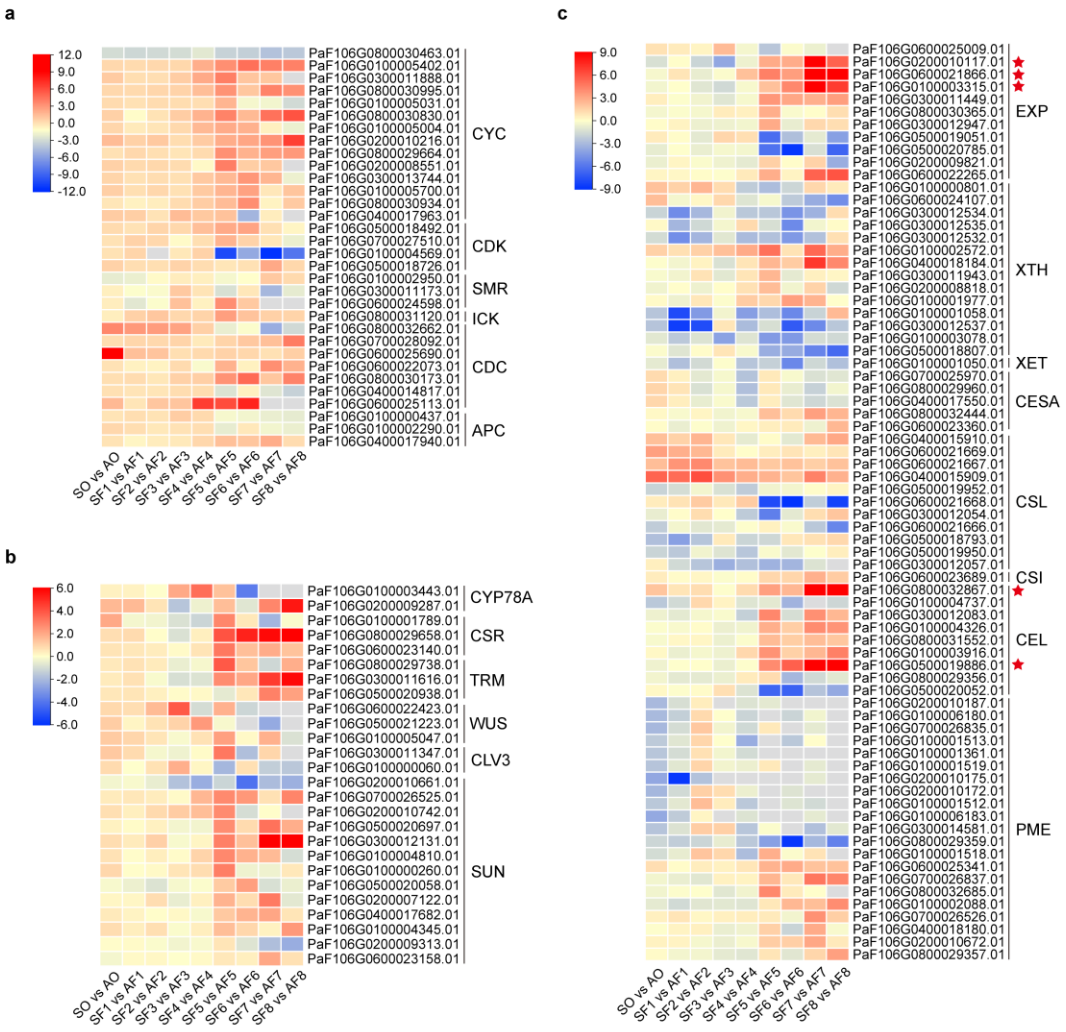

Figure S8. Heat maps showing the differences in expressions of underlying genes related to cell division and cell expansion. The redder color represents the higher log_2FC, and the bluer color represents the lower log_2FC. The yellow color represents the median value, and the gray color represents NA. **a**. The genes related to cell cycle control. **b**. The genes homologous to major known QTLs for fruit size-related traits. **c**. The genes related to cell wall loosening. The red stars indicate the genes with the most significant differences in gene expressions from the clustering branch of Figure 4d.

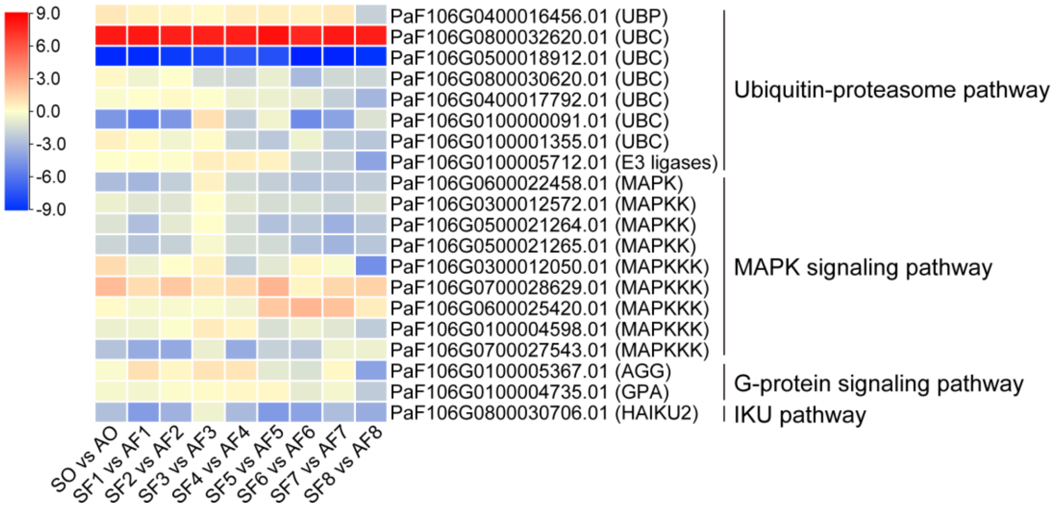

Figure S9. Heat map showing the differences in expressions of DEGs involved in signaling pathways. The redder color represents the higher log_2FC, and the bluer color represents the lower log_2FC. The yellow color represents the median value, and the gray color represents NA.

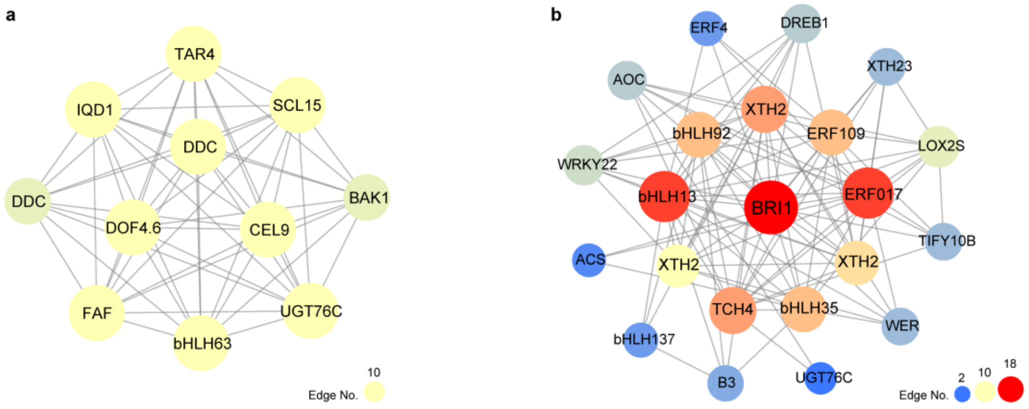

Figure S10. The co-expression networks of the MEgreenyellow module and the MEblack module. **a**. The co-expression network contained 11 co-expression genes in the MEgreenyellow module, and there were only 54 connected edges between these 11 genes. **b**. The co-expression network contained 22 co-expression genes in the MEblack module.

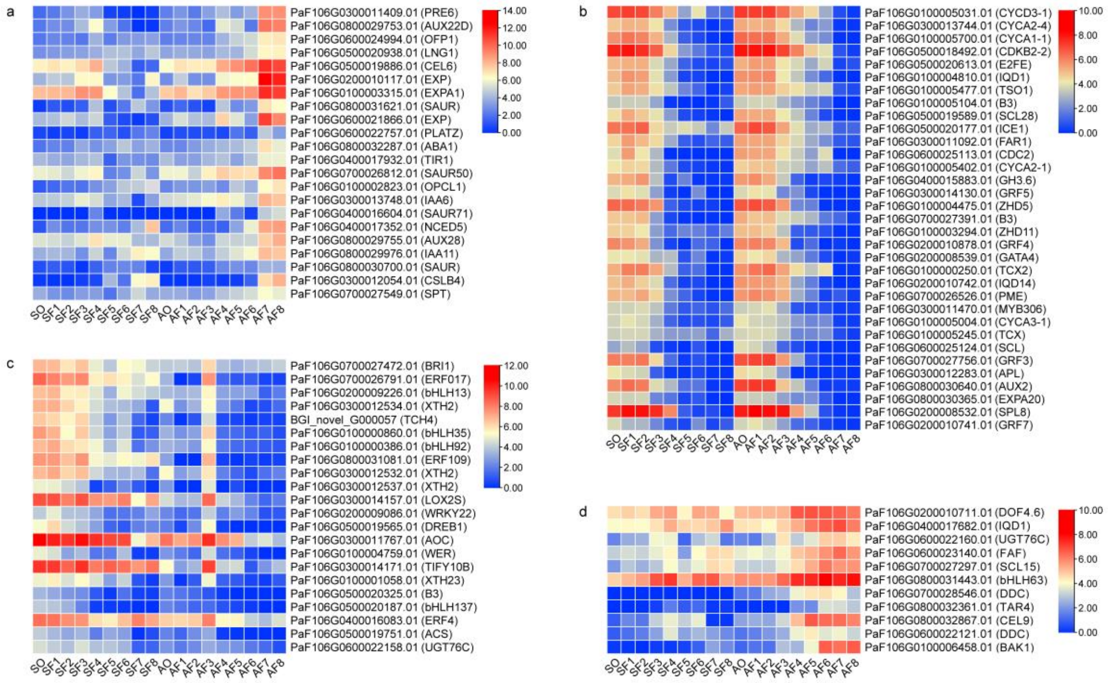

Figure S11. Heat maps showing the expressions of DEGs in the four gene co-expression networks. The expression levels of DEGs in all 18 samples from 'Sungold' and 'F43' were shown as $\log_2$(FPKM+1) values. The redder color represents the higher $\log_2$(FPKM+1), and the bluer color represents the lower $\log_2$(FPKM+1). The yellow color represents the median value. **a**. MEgreen module; **b**. MEturquoise module; **c**. MEblack module ; **d**: MEgreenyellow module.

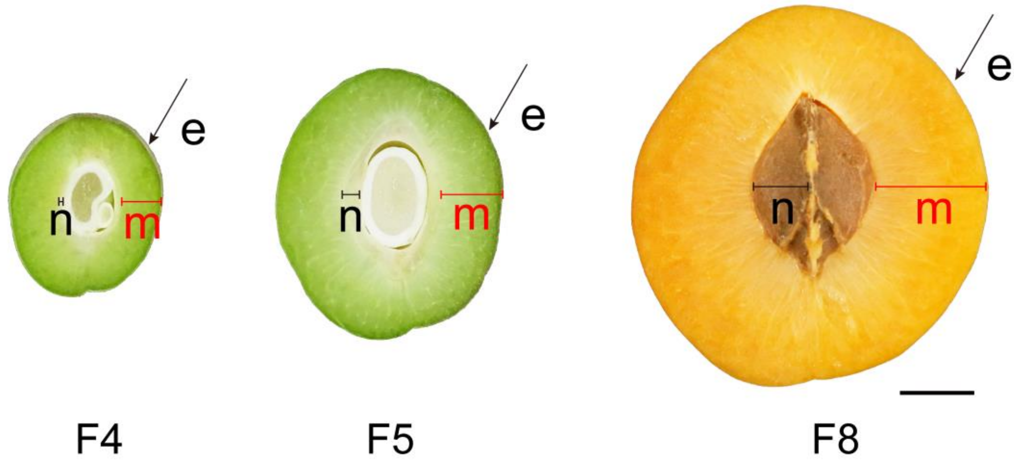

Figure S12. The diagram of the cross-section of the equatorial position of 'Sungold' fruit at stages F4, F5, and F8. The black arrows indicate the epicarp (e). The red line segments indicate the mesocarp (m) and the black line segments indicate the endocarp (n). bar = 1.0 cm. At stage F4, the endocarp is soft and can be cut directly; at stage F5, the endocarp is in the initial lignification process; at stage F8, the endocarp has been completely lignified.
